# Effect of Early Treatment With Hydroxychloroquine or Lopinavir and Ritonavir on Risk of Hospitalization Among Patients With COVID-19: The TOGETHER Randomized Clinical Trial

**DOI:** 10.1001/jamanetworkopen.2021.6468

**Published:** 2021-04-22

**Authors:** Gilmar Reis, Eduardo Augusto dos Santos Moreira Silva, Daniela Carla Medeiros Silva, Lehana Thabane, Gurmit Singh, Jay J. H. Park, Jamie I. Forrest, Ofir Harari, Castilho Vitor Quirino dos Santos, Ana Paula Figueiredo Guimarães de Almeida, Adhemar Dias de Figueiredo Neto, Leonardo Cançado Monteiro Savassi, Aline Cruz Milagres, Mauro Martins Teixeira, Maria Izabel Campos Simplicio, Luciene Barra Ribeiro, Rosemary Oliveira, Edward J. Mills

**Affiliations:** 1 Research Division, Cardresearch—Cardiologia Assistencial e de Pesquisa, Brazil; 2 Department of Medicine, Pontifícia Universidade Católica de Minas Gerais, Brazil; 3 Department of Health Research Methods, Evidence, and Impact, McMaster University, Hamilton, Ontario, Canada; 4 Department of Pathology and Molecular Medicine, McMaster University, Hamilton, Ontario, Canada; 5 Experimental Medicine, Department of Medicine, The University of British Columbia, Vancouver, British Columbia, Canada; 6 Cytel Inc, Vancouver, British Columbia, Canada; 7 Department of Public Health, Montes Claros State University, Montes Claros, Brazil; 8 Public Health Fellowship Program, Governador Valadares Public Health Authority, Brazil; 9 Public Health, Mental and Family Medicine Department, Ouro Preto Federal University, Ouro Preto, Brazil; 10 Public Health Care Division, City of Ibirité, Brazil; 11 Drug Research and Development Center, Federal University of Minas Gerais, Belo Horizonte, Brazil

## Abstract

**Importance:**

Data on the efficacy of hydroxychloroquine or lopinavir-ritonavir for the treatment of high-risk outpatients with COVID-19 in developing countries are needed.

**Objective:**

To determine whether hydroxychloroquine or lopinavir-ritonavir reduces hospitalization among high-risk patients with early symptomatic COVID-19 in an outpatient setting.

**Design, Setting, and Participants:**

This randomized clinical trial was conducted in Brazil. Recently symptomatic adults diagnosed with respiratory symptoms from SARS-CoV-2 infection were enrolled between June 2 and September 30, 2020. The planned sample size was 1476 patients, with interim analyses planned after 500 patients were enrolled. The trial was stopped after the interim analysis for futility with a sample size of 685 patients. Statistical analysis was performed in December 2020.

**Interventions:**

Patients were randomly assigned to hydroxychloroquine (800 mg loading dose, then 400 mg daily for 9 days), lopinavir-ritonavir (loading dose of 800 mg and 200 mg, respectively, every 12 hours followed by 400 mg and 100 mg, respectively, every 12 hours for the next 9 days), or placebo.

**Main Outcomes and Measures:**

The primary outcomes were COVID-19–associated hospitalization and death assessed at 90 days after randomization. COVID-19–associated hospitalization was analyzed with a Cox proportional hazards model. The trial included the following secondary outcomes: all-cause hospitalization, viral clearance, symptom resolution, and adverse events.

**Results:**

Of 685 participants, 632 (92.3%) self-identified as mixed-race, 377 (55.0%) were women, and the median (range) age was 53 (18-94) years. A total of 214 participants were randomized to hydroxychloroquine; 244, lopinavir-ritonavir; and 227, placebo. At first interim analysis, the data safety monitoring board recommended stopping enrollment of both hydroxychloroquine and lopinavir-ritonavir groups because of futility. The proportion of patients hospitalized for COVID-19 was 3.7% (8 participants) in the hydroxychloroquine group, 5.7% (14 participants) in the lopinavir-ritonavir group, and 4.8% (11 participants) in the placebo group. We found no significant differences between interventions for COVID-19–associated hospitalization (hydroxychloroquine: hazard ratio [HR], 0.76 [95% CI, 0.30-1.88]; lopinavir-ritonavir: HR, 1.16 [95% CI, 0.53-2.56] as well as for the secondary outcome of viral clearance through day 14 (hydroxychloroquine: odds ratio [OR], 0.91 [95% CI, 0.82-1.02]; lopinavir-ritonavir: OR, 1.04 [95% CI, 0.94-1.16]). At the end of the trial, there were 3 fatalities recorded, 1 in the placebo group and 2 in the lopinavir-ritonavir intervention group.

**Conclusions and Relevance:**

In this randomized clinical trial, neither hydroxychloroquine nor lopinavir-ritonavir showed any significant benefit for decreasing COVID-19–associated hospitalization or other secondary clinical outcomes. This trial suggests that expedient clinical trials can be implemented in low-income settings even during the COVID-19 pandemic.

**Trial Registration:**

ClinicalTrials.gov Identifier: NCT04403100

## Key Points

**Question:**

Does hydroxychloroquine or lopinavir-ritonavir, administered as a 9-day course, prevent COVID-19–associated hospitalization in patients with COVID-19?

**Findings:**

In this trial that included 685 patients, rates of COVID-19–associated hospitalization in patients treated with hydroxychloroquine or lopinavir-ritonavir were not significantly different compared with those who received placebo.

**Meaning:**

These findings may inform COVID-19 treatment guidelines for outpatients with COVID-19 and demonstrate that large-scale outpatient clinical trials of repurposed drugs can be successfully completed in low-income settings during the pandemic.

## Teaser

This randomized clinical trial examines whether hydroxychloroquine or lopinavir-ritonavir is effective for reducing hospitalization among high-risk outpatients with early symptomatic COVID-19.

## Introduction

SARS-CoV-2, the cause of COVID-19, has led to more than 109 million cases globally and more than 2.4 million deaths as of February 15, 2021. Brazil has been one of the most affected countries in the world, with more than 9.9 million infections that have caused more than 242 000 deaths.^1^

Unprecedented global research efforts to find effective treatments have identified remdesivir,^2^ shown to reduce the duration of hospitalization, and dexamethasone,^3^ shown to reduce mortality among severely sick patients. Although these treatments have reduced morbidity and mortality among hospitalized patients with COVID-19, developing treatments to prevent disease progression and hospitalization in an outpatient setting remain key priorities during the COVID-19 pandemic.^4,5,6^ In comparison with patients hospitalized with COVID-19, patients with COVID-19 in an outpatient setting will likely encompass different biological responses, so pharmaceutical interventions that show no clinical benefits in hospitalized settings may show different effects.^6^ Discovery of effective and affordable treatments for preventing disease progression and subsequent hospitalization in outpatient settings are critical to minimizing limited hospital resources, particularly for resource-limited settings.^5^ Repurposing existing treatments is an appealing approach, if effective, as they may already be available with known safety profiles.

Some repurposed drugs have demonstrated activity against coronaviruses in preclinical studies. Hydroxychloroquine has been reported to have in vitro activity against SARS-CoV-2^7,8^ through several mechanisms, including impairment of the terminal glycosylation of the angiotensin-converting enzyme receptor 2 (ACE2), the link between the envelope spike glycoprotein and also inhibiting the function of the endolysosome.^7^ Lopinavir-ritonavir, an HIV aspartate protease inhibitor type 1, has been reported to have in vitro inhibitory activity against SARS-CoV,^9^ and also improved clinical, radiological, and pathological outcomes in a marmoset model of Middle East respiratory syndrome coronavirus infection.^10^ Both drugs have been widely used in the pandemic, and evidence has now accumulated that they do not play a therapeutic role in advanced disease.^11,12^

Treating COVID-19 early in outpatient settings may be key to preventing progression of the disease.^13^ To evaluate the efficacy of hydroxychloroquine and lopinavir-ritonavir to prevent progression of COVID-19 and hospitalization among outpatients with laboratory-documented SARS-CoV-2, we conducted a randomized clinical trial in Brazil. This trial started with clinical evaluation of hydroxychloroquine and lopinavir-ritonavir using a common placebo control group. The flexible platform trial design was intended to allow for additional agents to be added and tested with standardized operating procedures outlined in a single overarching protocol called a master protocol.^14,15,16,17^

## Methods

### Study Design and Trial Oversight

The TOGETHER Trial is a randomized clinical trial to investigate the efficacy of repurposed treatments for SARS-CoV-2 infection among high-risk adult outpatients. The trial was designed and conducted in partnership with local public health authorities from 10 participating cities in Brazil. We enrolled participants starting on June 2, 2020, and concluded the trial on September 30, 2020. Informed participant consent was obtained orally by a trained member of the study staff team. The protocol was approved in compliance with the International Conference of Harmonization – Good Clinical Practices, as well as local regulatory requirements. The trial was approved for research ethics by local ethics board. The full protocol, statistical analysis plan, and additional details are listed in Supplement 1. We used the adaptive designs Consolidated Standards of Reporting Trials Extension (CONSORT Extension) (ACE) statement for reporting this trial.^18,19^

### Participants

This trial included participants if they were 18 years or older; reported less than 8 days since onset of flulike symptoms or chest computerized tomography scan consistent with COVID-19. Eligibility for participation also required at least one additional criterion for high risk: aged 50 years or older; presence of pulmonary disease, specifically moderate or severe persistent asthma, chronic obstructive pulmonary disease, pulmonary hypertension, or emphysema; diabetes requiring oral medication or insulin; hypertension requiring treatment; known cardiovascular diseases (congestive heart failure of any etiology, documented coronary artery disease, clinically manifest miscellaneous heart disease); symptomatic lung disease on chronic treatment; history of transplantation; obesity (body mass index ≥30 [calculated as weight in kilograms divided by height in meters squared]); immunocompromised status due to disease (eg, those living with HIV with a CD4 T-cell count of <200 cells/mm^3^, confirmed malignant neoplasm); immunocompromised status due to medication (eg, people taking 10 mg or more of prednisone equivalents a day); and patients with cancer.

Exclusion criteria were (1) use of any of study drugs in 30 days prior to screening; (2) clinical evidence of progression of COVID-19 (ie, use of oxygen supplementation; arterial oxygen saturation less than 94%; use of noninvasive positive-pressure ventilation support); (3) history of known life-threatening cardiac arrhythmias; (4) long QT syndrome; or (5) known allergy to study drugs.

### Setting

We involved the local public health authorities from participating cities in the research. This included facilitation of active partnerships with several levels of local public health structure in Brazil.

### Interventions and Randomization

We randomized patients to the hydroxychloroquine, lopinavir-ritonavir, and placebo groups at 1:1:1. Randomization was stratified by site, age (aged 50 years or older vs less than 50 years), and time of onset of flulike symptoms (at least 5 days vs less than 5 days). Patients, investigators, health care practitioners, and sponsors were masked to the study drug assignment. The randomization schedule was prepared by a masked statistician and provided to site-level pharmacists.

Patients assigned to the hydroxychloroquine group received a loading dose of 800 mg at the time of randomization and then 400 mg in daily doses at 8:00 am for 9 days. Patients assigned to lopinavir-ritonavir group received a loading dose of 800 mg of lopinavir and 200 mg of ritonavir at the first 2 intakes, followed by 400 mg of lopinavir and 100 mg of ritonavir every 12 hours for the next 9 days. Patients assigned to the placebo group received corresponding tablets of inert material (talc). Placebo bottles were matched for the same number of tablets as active hydroxychloroquine (placebo of hydroxychloroquine) and active lopinavir-ritonavir (placebo of lopinavir-ritonavir).

### Study Procedures

We presented the protocol to potential participants during phone calls at participating cities (daily epidemiological listing of suspected or confirmed cases of COVID-19 disease) and during medical consultation due to acute flulike symptoms. If participants agreed, we scheduled a home visit by physicians. After obtaining informed consent, the procedures for screening were performed and a swab sample was collected for SARS-CoV-2 reverse transcriptase–polymerase chain reaction (RT-PCR) testing in suspected cases and/or cases confirmed by other diagnostic methods. A randomization visit was done for patients with SARS-CoV-2–positive RT-PCR results. At the participant’s home, we performed all procedures at the same visit for patients who had already received a positive SARS-CoV-2 RT-PCR result within the last 72 hours. We assessed cardiac safety using a 6-lead electrocardiogram (ECG) (Kardiamobile), and patients with a corrected QT interval (QTc) of less than 480 milliseconds were randomized. We assessed oxygen status using a pulse oximeter for noninvasive arterial oxygen saturation (Spo_2_) and pulse (Jumper Medical Equipment), and temperature using a standard digital oral thermometer. Mid-turbinate nasal swab kits and sterile recipient storage were provided for nasal and oral samples as previously reported.^20^ On day 3, 7, 10, and 14, participants were instructed to self-record and transmit ECG for immediate analysis and also to collect nasal and oral samples for RT-PCR testing. Patients were contacted on a daily basis during the study drug period for medical adherence, adverse events, vital signs, and symptoms (Wisconsin Upper Respiratory Symptom Survey [WURSS-11]),^21,22^ and on days 14, 28, 56 and 90.

We performed RT-PCR testing that targeted the SARS-CoV-2 nucleocapsid genes N1 and N2.^23^ RT-PCR was performed on an ABI 7500 real-time PCR system (Applied Biosystems). An internal control amplification, RNase P, a human gene marker, was performed to monitor RNA extraction and RT-PCR quality. Specimens were considered positive if either or both N1 and N2 targets were detected. The cycle threshold, a semiquantitative measure of viral load, was less than or equal to 38.

### Outcome Measures

Our primary outcomes were COVID-associated hospitalization and death. We assessed the primary outcomes at 90 days after randomization. Secondary end points were hospital admissions for any cause; proportion of persons with clearance of SARS-CoV-2 (defined as 1 negative swab since baseline) at day 3, 7, and 14; and treatment-emergent adverse events.

All serious and nonserious adverse events were reported as per local regulatory requirements. Reportable adverse events (AEs) included serious AEs, AEs resulting in study medication discontinuation, and AEs assessed related to study medication. QTc was monitored via 6-lead ECG at prespecified days after randomization. The digital recordings were deidentified and transferred to a central facility (Cardresearch) for reading. The QT intervals were measured by a certified cardiologist using the Bazett formula with a combination of automated detection of fiducial points with manual adjustment using a novel interface that allowed review of all 6 leads separately as well as calculated median beats to minimize erroneous measurements. If QTc was greater than 500 milliseconds or greater than 60 milliseconds above the baseline reading, study medication was withheld, and the ECG was repeated. Study drugs were discontinued if a second ECG confirmed a QTc change.

### Statistical Analyses

#### Sample Size

A sample size of 492 patients per group was chosen for each experimental group to achieve 80% power with α = .05, 2-sided type 1 error for a pairwise comparison against the control (talc) to detect minimum treatment efficacy defined by 37.5% relative risk reduction of preventing hospitalization assuming a control event rate of 20%. According to these assumptions, 160 events per pairwise comparison would be needed: 98 controls. Although the sample size calculation was done based on a binary outcome, the protocol dictated that COVID-19 hospitalization and COVID-19–associated mortality (primary outcomes) be analyzed using time-to-event analysis (ie, Cox regression analysis) if the proportional hazards assumption was met. Otherwise, it was prespecified that these primary outcomes would be analyzed as binary.

#### Analyses

Baseline characteristics are reported as proportions or median and interquartile range (IQR) for continuous variables. The Cox proportional hazards model was used for the analysis of time-to-event outcomes of COVID-19–associated and all-cause hospitalizations for both intention-to-treat (ITT) and per-protocol (PP) analyses.

For viral clearance we fitted a longitudinal, mixed-effect logistic regression model with a treatment and time interaction term for binary patient outcomes (COVID-19 positive or negative) reported on day 3, 7, and 14 from randomization, with participant random effect. PP analyses were considered sensitivity analyses to assess the robustness of the results. The criterion for statistical significance was set at α = .05. All analyses were performed using R version 3.6.3 (R Project for Statistical Computing) in December 2020.

#### Data Safety Monitoring

A data safety monitoring board (DSMB) was instituted to provide independent oversight for the trial. An interim analysis for early stopping based on either superiority or futility was planned to be conducted with data cut from September 18, 2020. At that time, a total of 500 patients were expected to have been enrolled into the trial, with 400 patients (approximately 133 per group) having undergone adequate follow-up to observe the outcome of hospitalization.

## Results

### Participants

**Figure 1.**
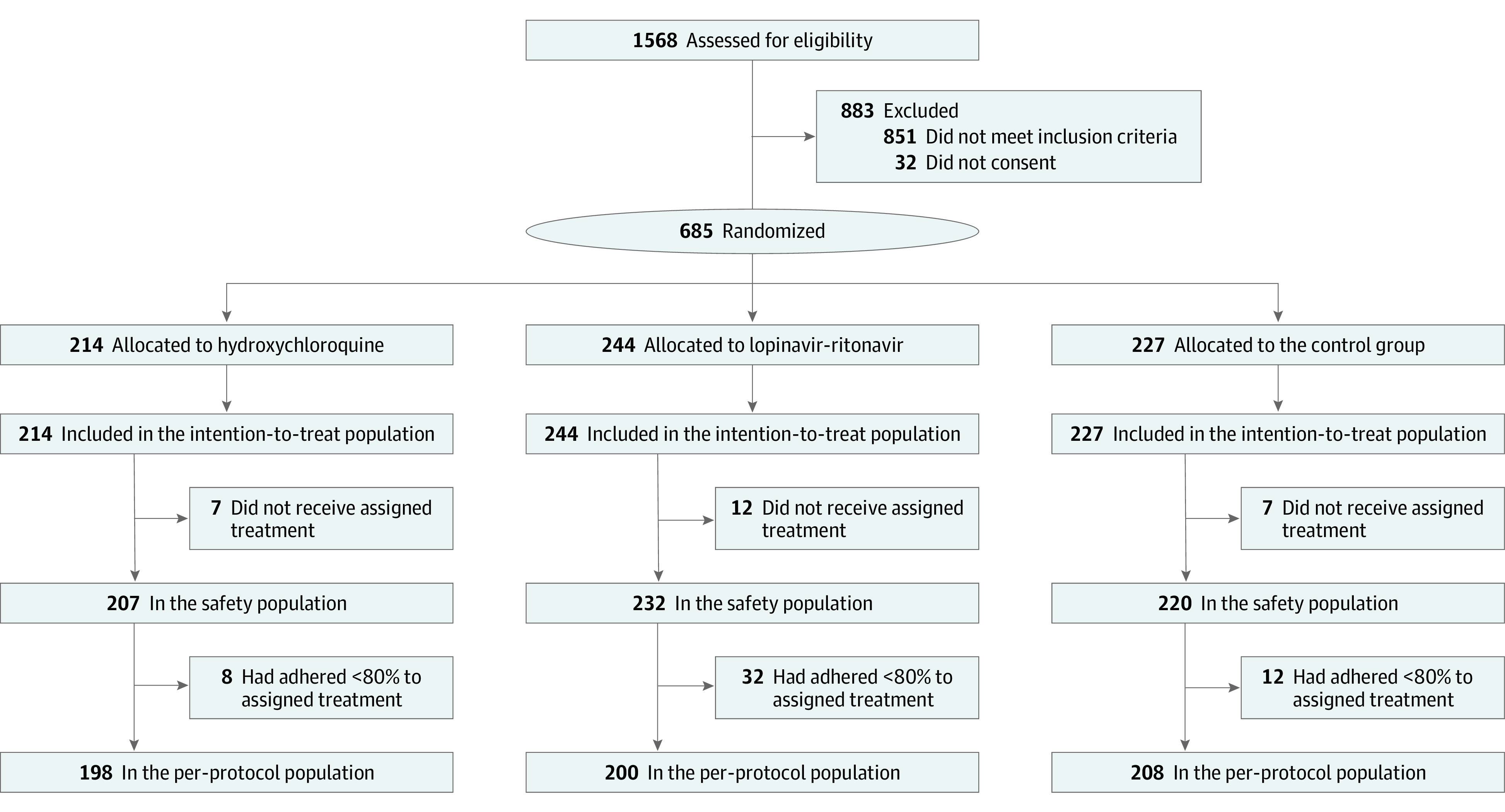
Flow Diagram of Participants in the TOGETHER Trial

Between June 2 and October 9, 2020, 685 participants were recruited and randomized. Among these participants, 632 (92.3%) self-identified as mixed-race, 377 (55.0%) were women, and the median (range) age was 53 (18-94) years (Table 1). In total, 214 participants were allocated to the hydroxychloroquine group, 244 were allocated to the lopinavir-ritonavir group, and 227 to the placebo group (Figure 1). With respect to covariates of age, body mass index, and comorbidities, the groups were generally well balanced (Table 1). At the end of the trial, 79 participants (11.5%) did not complete all phases of the study. The lopinavir-ritonavir intervention group had 44 participants (18%) who did not complete the study, which was more than either of the other 2 groups (Figure 1).

**Table 1.** Patient Characteristics by Treatment Allocation in the TOGETHER Trial

Characteristic	Participants, No. (%)			
	Hydroxychloroquine (n = 214)	Lopinavir-ritonavir (n = 244)	Placebo (n = 227)	Total (N = 685)
Sex				
Female	122 (57.0)	134 (54.9)	121 (53.3)	377 (55.0)
Male	92 (43.0)	110 (45.1)	106 (46.7)	308 (45.0)
Race				
Mixed-race^a^	204 (95.3)	223 (91.4)	205 (90.3)	632 (92.3)
White	4 (1.9)	6 (2.5)	8 (3.5)	18 (2.6)
Black or African American	2 (0.9)	6 (2.5)	5 (2.2)	13 (1.9)
Other^b^	0	0	2 (0.9)	2 (0.3)
Unknown	4 (1.9)	9 (3.7)	7 (3.1)	20 (2.9)
Age, y				
Median (IQR)	53 (18-81)	54 (18-94)	53 (18-80)	53 (18-94)
>50	127 (59.3)	140 (57.4)	135 (59.5)	402 (58.7)
BMI^c^				
Median (IQR)	28.8 (25.1-32.3)	29.2 (25.6-32.9)	28.1 (25.2-32.9)	28.7 (25.4-32.6)
<30	125 (58.4)	128 (52.5)	137 (60.4)	390 (56.9)
≥30	88 (41.1)	109 (44.7)	89 (39.2)	286 (41.8)
Unspecified	1 (0.5)	7 (2.9)	1 (0.4)	9 (1.3)
Time since onset of symptoms, d				
>5	177 (82.7)	210 (86.1)	187 (82.4)	574 (83.8)
≤5	37 (17.3)	34 (13.9)	40 (17.6)	111 (16.2)
Risk factors				
Chronic cardiac disease	6 (2.8)	13 (5.3)	8 (3.5)	27 (3.9)
Hypertension	101 (47.2)	128 (52.5)	109 (48.0)	338 (49.3)
Chronic pulmonary disease	7 (3.3)	4 (1.6)	6 (2.6)	17 (2.5)
Asthma	24 (11.2)	15 (6.1)	20 (8.8)	59 (8.6)
Chronic kidney disease	1 (0.5)	1 (0.4)	3 (1.3)	5 (0.7)
Mild liver disease	0 (0.0)	0 (0.0)	2 (0.9)	2 (0.3)
Rheumatologic disorder	4 (1.9)	2 (0.8)	3 (1.3)	9 (1.3)
Chronic neurological disorder	1 (0.5)	0 (0.0)	0 (0.0)	1 (0.1)
Diabetes				
Type 1	17 (7.9)	13 (5.3)	13 (5.7)	43 (6.3)
Type 2	24 (11.2)	31 (12.7)	35 (15.4)	90 (13.1)
Obesity	70 (32.7)	83 (34.0)	81 (35.7)	234 (34.2)
Malignant neoplasm	2 (0.9)	2 (0.8)	4 (1.8)	8 (1.2)
HIV/AIDS	1 (0.5)	0 (0.0)	6 (2.6)	7 (1.0)
Autoimmune disease	1 (0.5)	3 (1.2)	1 (0.4)	5 (0.7)
Smoking	12 (5.6)	12 (4.9)	11 (4.8)	35 (5.1)
Any other risk factor(s)	8 (3.7)	8 (3.3)	9 (4.0)	25 (3.6)
Participants with multiple risk factors^d^	121 (56.5)	134 (54.9)	134 (59.0)	389 (56.8)
Risk factors per participant, median (IQR), No.	1.0 (1.0-2.0)	1.0 (1.0-2.0)	1.0 (1.0-2.0)	1.0 (1.0-2.0)
Symptoms				
Chest tightness	29 (13.6)	47 (19.3)	35 (15.4)	111 (16.2)
Dry cough	64 (29.9)	70 (28.7)	59 (26.0)	193 (18.2)
Sore throat	63 (29.4)	59 (24.2)	59 (26.0)	181 (26.4)

Abbreviations: BMI, body mass index; IQR, interquartile range.

^a^ Self-identified as someone with mixed-race ancestry.

^b^ Other included non-White, non-Black, non-Hispanic Brazilians.

^c^ BMI is calculated as weight in kilograms divided by height in meters squared.

^d^ Includes congenital heart disease.

### Primary Outcomes

**Figure 2.**
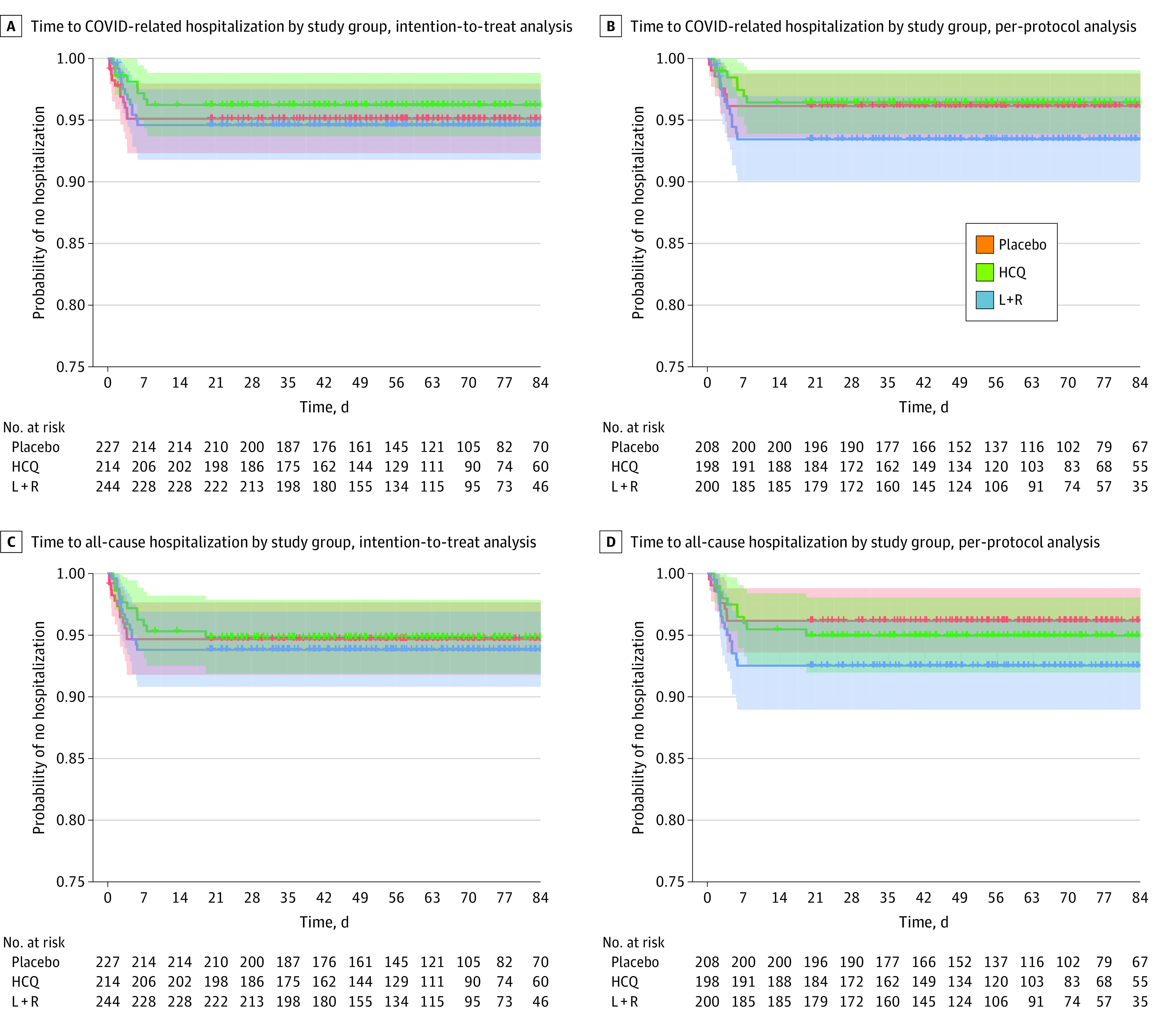
Time to Hospitalization by Study Group Shaded areas indicate 95% CIs; HCQ, hydroxychloroquine; and L + R, lopinavir-ritonavir.

At the end of the trial, 8 participants from the hydroxychloroquine group (3.7%) had a COVID-19–associated hospitalization, and the median (IQR) time from randomization to hospitalization was 4.8 (1.4-6.1) days; 14 participants from the lopinavir-ritonavir group (5.7%) had a COVID-19–associated hospitalization, and the median (IQR) time from randomization to hospitalization was 3.6 (2.5-4.8) days; and 11 participants from the control group (4.8%) had a COVID-19–associated hospitalization, and the median (IQR) time from randomization to hospitalization was 2.4 (0.8-3.2) days (Table 2). In the Cox model, there was no statistically significant difference in the risk of hospitalization between the 2 intervention groups and the placebo group (Table 2, Figure 2; eTable 1 in Supplement 2). For the hydroxychloroquine group, the hazard ratio (HR) was 0.76 (95% CI, 0.30-1.88) based on ITT analysis and 0.91 (95% CI, 0.33-2.52) based on PP analysis for COVID-19–associated hospitalization outcome; for the lopinavir-ritonavir group, the HR was 1.16 (95% CI, 0.53-2.56) based on ITT analysis and 1.82 (95% CI, 0.76-4.35) based on PP analysis for COVID-19–associated hospitalization outcome. At the end of the trial, we recorded 3 fatalities, 1 in the placebo group and 2 in the lopinavir-ritonavir intervention group.

**Table 2.** Hospitalization Primary Outcome by Intention-to-Treat and Per-Protocol Analysis

Outcome	Intention-to-treat analysis					Per-protocol analysis				
	Participants, No.	Hospitalized, No. (%)	Time to hospitalization, median (IQR), d		Effect (95% CI)	Participants, No.	Hospitalized, No. (%)	Time to hospitalization, median (IQR), d		Effect (95% CI)^a^
COVID-19 hospitalization										
Hydroxychloroquine	214	8 (3.7)	4.8 (1.40-6.12)		0.76 (0.30-1.88)^b^	198	7 (3.5)	5.8 (2.63-6.43)		0.91 (0.33-2.52)^b^
Lopinavir-ritonavir	244	14 (5.7)	3.6 (2.50-4.76)		1.16 (0.53-2.56)^b^	200	14 (7)	3.6 (2.50-4.76)		1.82 (0.76-4.35)^b^
Placebo	227	11 (4.8)	2.4 (0.76-3.20)		1 [Reference]	208	8 (3.8)	2.4 (1.25-3.46)		1 [Reference]
All	685	33 (4.8)	3.0 (1.43-4.75)		NA	606	29 (4.8)	3.4 (2.25-4.76)		NA
All-cause hospitalization										
Hydroxychloroquine	214	11 (5.1)	3.8 (1.88-6.43)		0.96 (0.42-2.17)^b^	198	10 (5.1)	4.8 (2.48-6.73)		1.31 (0.52-3.31)^b^
Lopinavir-ritonavir	244	16 (6.6)	3.1 (2.31-4.75)		1.22 (0.58-2.57)^b^	200	16 (8)	3.1 (2.31-4.75)		2.08 (0.89-4.87)^b^
Placebo	227	12 (5.3)	2.2 (0.79-3.12)		1 [Reference]	208	8 (3.8)	2.4 (1.25-3.46)		1 [Reference]
All	685	39 (5.7)	2.9 (1.83-4.56)		NA	606	34 (5.6)	3.1 (2.27-4.76)		NA
Time to viral clearance										
Hydroxychloroquine	185	97 (52.4)		NA	0.92 (0.82-1.02)^c^	179	102 (57.0)		NA	0.98 (0.87-1.09)^c^
Lopinavir-ritonavir	201	125 (62.2)		NA	1.04 (0.94-1.16)^c^	182	111 (61.0)		NA	1.01 (0.91-1.13)^c^
Placebo	195	112 (57.4)		NA	1 [Reference]	181	102 (56.4)		NA	1 [Reference]
All	581	334 (57.5)		NA	NA	542	315 (58.1)		NA	NA

Abbreviations: IQR, interquartile range; NA, not applicable.

^a^ The hazard ratio (and 95% CI) for each of the experimental groups in comparison to the placebo group were determined using Cox proportional hazards model. The odds ratios presented here were determined using longitudinal, mixed-effect logistic regression models with a treatment and time interaction term for binary patient outcomes (COVID-19 positive or negative) reported on day 3, 7, and 14 from randomization, with participant random effect.

^b^ Values are hazard ratios.

^c^ Values are odds ratios.

### Virological Clearance

In the mixed-effect logistic model, the clearance for the hydroxychloroquine (odds ratio [OR], 0.91; 95% CI, 0.82-1.02) and lopinavir-ritonavir (OR, 1.04; 95% CI, 0.94-1.16) groups did not differ in comparison with the control group (Table 2; eTable 3 in Supplement 2). Similar to hospitalization outcomes, neither hydroxychloroquine nor lopinavir-ritonavir showed difference in viral clearance across all prespecified subgroups based on ITT analysis (eTable 4 in Supplement 2).

### Symptom Resolution

We found no difference in the resolution of combined symptoms using the WURSS scale between either hydroxychloroquine and placebo or lopinavir-ritonavir and placebo, or for individual symptoms. For time to chest tightness symptom resolution, hydroxychloroquine had an HR of 1.39 (95% CI, 0.87-2.22) and lopinavir-ritonavir had an HR of 0.99 (95% CI, 0.65-1.51) based on the ITT analysis. For time to dry cough resolution, ITT analysis showed an HR of 0.86 (95% CI, 0.57-1.29) for hydroxychloroquine and an HR of 0.76 (95% CI, 0.51-1.13) for lopinavir-ritonavir. For time to sore throat symptom resolution, hydroxychloroquine had an HR of 1.11 (95% CI, 0.81-1.53) and lopinavir-ritonavir had an HR of 0.88 (95% CI, 0.64-1.22) based on ITT analysis. The PP analyses on time to symptom resolution were similar to the ITT analysis.

### Adverse Events

There were 46 (22.2%) treatment-emergent adverse events in the hydroxychloroquine group, of which 11 (5.3%) were serious and 1 (0.5%) leading to discontinuation. In the lopinavir-ritonavir group, there were 92 (39.7%) treatment-emergent adverse events, of which 20 (8.6%) were serious and 0 leading to discontinuation. In the placebo group, we recorded 46 (20.9%) treatment-emergent adverse events, of which 12 were serious (5.5%) and 0 leading to discontinuation (Table 3).

**Table 3.** Treatment-Emergent Adverse Events in the TOGETHER Trial

Outcome	Patients, No. (%)			
	Hydroxychloroquine (n = 207)	Lopinavir-ritonavir (n = 232)	Placebo (n = 220)	Total (n = 659)
Any TEAE	46 (22.2)	92 (39.7)	46 (20.9)	184 (27.9)
Serious TEAE	11 (5.3)	20 (8.6)	12 (5.5)	43 (6.5)
TEAE leading to withdrawal of study drug	1 (0.5)	9 (3.9)	3 (1.4)	13 (2.0)
TAEA leading to study termination	1 (0.5)	0	0	1 (0.2)
TEAEs by severity				
Grade 1	30 (14.5)	69 (29.7	33 (15.0)	132 (20.0)
Grade 2	6 (2.9)	6 (2.6)	2 (0.9)	4 (2.1)
Grade 3	9 (4.3)	13 (5.6)	7 (3.2)	29 (4.4)
Grade 4	1 (0.5)	2 (0.9)	3 (1.4)	6 (0.9)
Grade 5	0	2 (0.9)	1 (0.5)	3 (0.5)
Noncardiac disorder TEAEs by severity				
Grade 1	30 (14.5)	69 (29.7)	31 (14.1)	130 (19.7)
Grade 2	6 (2.9)	6 (2.6)	2 (0.9)	14 (2.1)
Grade 3	9 (4.3)	13 (5.6)	7 (3.2)	29 (4.4)
Grade 4	1 (0.5)	2 (0.9)	3 (1.4)	6 (0.9)
Grade 5	0	2 (0.9)	1 (0.5)	3 (0.5)
Cardiac disorder TEAEs by severity				
Grade 1	0	1 (0.4)	2 (0.9)	3 (0.5)
Grade 2	0	0	0	0
Grade 3	0	0	0	0
Grade 4	0	0	0	0
Grade 5	0	0	0	0

Abbreviation: TEAE, treatment-emergent adverse event.

Our study discontinued the treatment groups because of a low number of events occurring among participants in each group. We conducted a sample size reestimation using bayesian predictive power based on the actual event rate occurring in our trial and detected that greater than 10 000 participants would be required to detect a statistically significant difference between groups, assuming the event rate remained constant.^24^ The DSMB recommended discontinuing the trial as this would be clinically irrelevant for recommending either treatment.

## Discussion

Our study found no significant differences among hydroxychloroquine, lopinavir-ritonavir, or placebo in COVID-19–associated hospitalizations, time to hospitalization, or time to viral clearance. For both ITT and PP analyses of these clinical outcomes for the entire trial cohort and the subgroups, no important clinical benefits of hydroxychloroquine or lopinavir-ritonavir were found. The independent DSMB, based on interim analysis results, made the decision to stop enrollment to the hydroxychloroquine and lopinavir-ritonavir groups because of a low number of emerging events. This study reports on the final results inclusive of patients who had been randomized to hydroxychloroquine or lopinavir-ritonavir between the time of data-cut for interim analysis to the time of the DSMB meeting.

During this pandemic, some drugs received great interest because of preclinical studies showing a potential benefit both in controlling viral replication and in eradicating both SARS-CoV and SARS-CoV-2, including experimental models of infection. In this context, repurposed therapies appear as an important option in the context of an emerging disease with no treatment option to date and high morbidity and mortality.^25^ In addition, their low cost, widespread availability, and known safety profiles make these drugs ideal in the context of COVID-19. Unfortunately, no repurposed therapies have been effective in studies of moderate to severe forms of the disease, so all current efforts have been focused on evaluating these options in patients with mild, early disease, arguing that the control of viral load in this phase will minimize the development of more serious COVID-19.^13^ Nevertheless, this discussion went beyond the frontiers of science and acquired political and ideological contours, which brought additional difficulty in conducting these studies.^26^ Brazil was particularly influenced by this bias, which resulted in the dismissal of 2 ministers of health and a wide-ranging social media campaign in favor of using treatments in the early stages of COVID-19 without proper proof of its effectiveness. In this context, this trial was initiated. We decided to use an adaptive clinical trial design to provide a faster response, through a centralized randomization system, using quadruple masking, and independent data analysis, and following CONSORT recommendations for clinical trials.

We chose to evaluate hydroxychloroquine and lopinavir-ritonavir because of the early and widescale interest in their treatment effects and their off-label use early on in the pandemic. Much has been written about the roles of these drugs in the treatment of COVID-19 and the scientific debate and political support that hydroxychloroquine, in particular, received.^26^ During the conduct of our trial, data emerged on the role of both hydroxychloroquine or lopinavir-ritonavir among hospitalized patients via the RECOVERY trial. For both drugs, it became clear that they did not play a role in patients with advanced disease who were hospitalized.^11,27^ Similarly, there has been no demonstrated efficacy shown for hydroxychloroquine in outpatient populations.^28,29^ There had been, however, equipoise as to whether these drugs would play a role in prophylaxis and early treatment of COVID-19 infection.^30^ Our study, among the largest trial of these drugs in early treatment, suggests that hydroxychloroquine and lopinavir-ritonavir do not have a role in early treatment of COVID-19.

### Strengths and Limitations

Our study has both strengths and limitations. Strengths include the identification and rapid recruitment of high-risk patients for developing severe COVID-19. We used a network of local primary care centers in Brazil and sought the political support of local leaders to endorse the trial and encourage the population to participate. We successfully recruited patients in the primary care settings and made home visits using appropriate personal protective equipment by physicians and medical students. We experienced low loss-to-follow up of participants in the trial. From the first patient recruited to the completion of the enrollment for active interventions of hydroxychloroquine and lopinavir-ritonavir took just 90 days. To our knowledge, in addition to ANTICOV recently launched by the Drugs for Neglected Diseases Initiatives (DNDI) for several sub-Saharan African countries to study treatments in mild-to-moderate patients with COVID-19, this trial in Brazil is one of the first large randomized clinical trials for repurposed drugs that is being conducted solely in the setting of low- and middle-income countries. Although this publication reports the clinical findings of hydroxychloroquine and lopinavir-ritonavir group in Brazil only, we had extended this trial to South Africa and a related trial in the United States. Given the DSMB decision, based on the Brazilian data confirming a lack of clinical benefits for hydroxychloroquine and lopinavir-ritonavir for outpatient treatment of patients with COVID-19, the enrollment in the United States and South Africa for these interventions has been discontinued. Other agents are rapidly being screened and developed for SARS-CoV-2 infection and can be incorporated into this master protocol for an outpatient platform trial as additional groups. The flexible platform trial design allows additional agents to be added and tested with standardized eligibility criteria, outcomes, and measurements. If an intervention is shown to be effective, this design allows the replacement of the placebo group with the effective intervention as the comparator.

Limitations of our trial were predominantly related to the challenges associated with conducting a trial during a pandemic wherein the disease was not well understood. We still do not understand the best clinical outcome to evaluate in trials for early treatment and prophylaxis trials. It is possible that neither viral clearance or hospitalization are the most important outcome, and outcomes such as inflammatory response may be more important. We found a low rate of hospitalizations, even though our population had risk factors for developing serious COVID-19 and median (range) age of 53 (18-94) years. It seems likely that we do not yet understand what demographic characteristics are associated with the highest risk of developing severe COVID-19.

Repurposing existing drugs is an appealing strategy to respond to the pandemic.^31^ As of the writing of this study, as many as 388 clinical trials have been registered evaluating hydroxychloroquine or lopinavir-ritonavir. Several early treatment and postexposure trials have been completed evaluating hydroxychloroquine and none have demonstrated important treatment effects.^29,32^ Although consensus is emerging that hydroxychloroquine and lopinavir-ritonavir do not confer any benefit for treating COVID-19, evaluating other repurposed drugs is still warranted. The ANTICOV^33^ and TREATNOW are 2 trials registered currently evaluating hydroxychloroquine and lopinavir-ritonavir, respectively, in outpatient populations.^34^ Our findings from this large trial in an outpatient population to examine the efficacy of hydroxychloroquine or lopinavir-ritonavir suggest that the trial infrastructure from those trials may be more appropriately designed to evaluate other repurposed drugs for which there is little experimental evidence to evaluate their efficacy. Our study group is now evaluating the effect of ivermectin, fluvoxamine, and metformin on COVID-19–associated hospitalizations.

## Conclusions

This randomized clinical trial found no clinical benefit to support the use of either hydroxychloroquine or lopinavir-ritonavir in an outpatient population. This adds to the growing evidence that these drugs should not be used for the treatment of COVID-19. While evidence emerges to evaluate these drugs as prophylaxis, as treatment for both outpatients and inpatients, hydroxychloroquine and lopinavir-ritonavir do not appear to confer any clinical benefit. These results might affect several countries deciding whether to continue to offer both drug regimens for ambulatory patients presenting with mild COVID-19. The successful completion of this trial also demonstrates that expedient trials of repurposed drugs can be completed in low-income settings even during a global health crisis such as the COVID-19 pandemic.

## Notes

Supplement 1. **Trial Protocol**

Supplement 2. **eAppendix.** Trial Organization **eTable 1. **Summary of Cox Proportional Hazard Regression Model for COVID-19 And All-Cause Hospitalization **eTable 2.** Summary of Sub-Group Analyses for COVID-19 And All-Cause Hospitalization Outcomes **eTable 3.** Summary of Mixed-Effect Logistic Regression Model for Viral Suppression **eTable 4.** Summary of Sub-Group Analyses on Viral Suppression, Intention to Treat Analysis **eTable 5.** Summary of Cox Proportional Hazard Regression Model for Time to COVID-19 Related Symptoms Resolution

Supplement 3. **Data Sharing Statement**
