# Error in Visual Abstract

**DOI:** 10.1001/jamanetworkopen.2021.30442

**Published:** 2021-09-17

In the Original Investigation titled “Effect of Early Treatment With Hydroxychloroquine or Lopinavir and Ritonavir on Risk of Hospitalization Among Patients With COVID-19: The TOGETHER Randomized Clinical Trial,”^1^ published April 22, 2021, there were errors in the visual abstract. The graph labels for placebo and hydroxychloroquine were mistakenly transposed, the hazard ratio for lopinavir and ritonavir should have been 1.16 instead of 1.17, and the Population section should not have mentioned expected hospital stays. This article has been corrected.^1^
