# Supplementary material for: Effect of Early Treatment With Hydroxychloroquine or Lopinavir and Ritonavir on Risk of Hospitalization Among Patients With COVID-19: The TOGETHER Randomized Clinical Trial
Source: JAMA Netw Open. 2021 Apr 22;4(4):e216468. doi: 10.1001/jamanetworkopen.2021.6468 (PMC8063069; doi:10.1001/jamanetworkopen.2021.6468)
Supplement: Supplement 1. — Trial Protocol [file jamanetwopen-e216468-s001.pdf]

TOGETHER Trial: An Adaptive Randomized Platform Trial to Investigate the Efficacy of Novel Agents for Treatment of SARS-CoV-2 Infection Among High-Risk Outpatient Adults

Study code	Early Tx for SARS-CoV-2
Short title	Outpatient Management of SARS-CoV-2 for High-Risk Adults
Initial investigational products and comparator for Brazil (TOGETHER 2)	• Lopinavir/ritonavir (LPV/r)<br>• Hydroxychloroquine (HCQ)<br>• Placebo pills
Other potential investigational products	Other chloroquine-based therapies, other antivirals, and other candidate regimens
Date of protocol	29 July 2020
Protocol version	3.0
Protocol registry number	NCT04403100 (TOGETHER 2)<br>TBD (TOGETHER 3)
Summary of revision history for TOGHETER 2	Not applicable
Funder	Bill and Melinda Gates Foundation through the University of Washington

Protocol amendments (Version 2 to Version 3)

Previous protocol	New protocol
Version 2<br>Date: 18 June 2020	Version 3<br>Date: 28 July 2020

Inclusion criteria

Version 2

Not applicable

Version 3

Addition

COVID-19 symptom onset of less than 8 days with the day of onset of symptoms counted as Day 0

Reason

This is in response to the CTC discussion and written recommendation dated 27 July 2020 to only include participants with early disease.

The population who may benefit from early antiviral therapy are those who are ambulatory but may progress to requiring hospitalisation while in self-isolation. This population is likely to include individuals with underlying co-morbidities as outlined in our inclusion criteria. However, we feel that it is still important to include a smaller group of low-risk participants in our study to learn more about natural history. *Treatment as early as possible after symptom onset is likely an important predictor of an antiviral efficacy to prevent disease progression.* The more difficult assessment is when antiviral therapy is too late, most likely when the inflammatory components of COVID-19 disease predominate. We made the following assumptions:

- Our primary endpoint is the development of lower respiratory tract symptoms defined as SpO_2<93% or decline from baseline of 6% in 2 measurements at least 2 hours apart. We assume that our participant population will deteriorate based on their co-morbidities and risk factor profile and reviewed when viral burden is expected to reach maximum levels in a hospitalised population.
- The highest detection of SARS-CoV-2 RNA in serum and throat samples are between 11 to 15 days after symptom onset in hospitalised patients.[1] Others also support that viral load shedding peaks around 15 days in hospitalised patients.[2]
- We conservatively took 11 days as the viral peak and concluded that at minimum, a 5 day course should be administered prior to the viral peak based on oseltamivir experience for influenza. Therefor the last day of treatment initiation after symptom onset should be day 7.

- Initiating treatment from day 7 onwards for 5 days will take us to day 11 from symptom onset. We therefore set the inclusion criteria that participants should only be enrolled and initiated on antiviral therapy less than 8 days from symptom onset.

[1] Xu D, Zhou F, Sun W, et al. Relationship Between serum SARS-CoV-2 nucleic acid(RNAemia) and Organ Damage in COVID-19 Patients: A Cohort Study [published online ahead of print, 2020 Jul 28]. Clin Infect Dis. 2020;ciaa1085. doi:10.1093/cid/ciaa1085

[2] Liu Y, Yan LM, Wan L, et al. Viral dynamics in mild and severe cases of COVID-19. Lancet Infect Dis. 2020;20(6):656-657. doi:10.1016/S1473-3099(20)30232-2

7.10 Additional treatment arm selection in future protocol amendments

Version 2

Not applicable

Version 3

Addition

The TOGETHER Consortium is a multi-site platform trial to evaluate affordable and scalable outpatient treatment options. The flexible platform trial design will allow additional agents to be added into the trial. Therefore, this will not be a randomised clinical trial solely focused on the current protocol interventions, and new interventions will be added later into the trial based on new emerging evidence. Any amendments require regulatory and ethical approval prior to implementation. The future intervention selection will be through the COVID-19 Therapeutics Accelerator initiative funded by the Bill and Melinda Gates Foundation (BMGF).

The COVID-19 Therapeutics Accelerator is supported by a multi-disciplinary group of clinicians and scientists who provide heuristic input on intervention selection based on clinical safety, clinical pharmacology and suitability for low middle-income countries; considerations such as ease of use, adherence and manufacturing and scale up all influence the selection of regimens to study. The COVID-19 Therapeutics Accelerator further examines activity of putative antivirals in various cell lines including VERO E6 cells, human lung epithelial cells, CALU-3 human lung cancer cells and others. Examination of both activities alone and also in combination therapy is under way. Therapy regimens are also guided by in silico assessments using viral cell cycle model and translational approaches including physiological based PK modelling, to contextualize the concentrations in vitro to what might be achieved in vivo.

The decision to add new interventions into the TOGETHER Consortium will be facilitated by Cytel who regularly engage with the BMGF and the COVID-19 Therapeutics Accelerator. With funding from the BMGF, Cytel has created a real-time global COVID-19 clinical trial tracker (www.covid19-trials.org) to collate all clinical trials registered across several global clinical trial registries. For the TOGETHER Consortium, this trial tracker will collate all clinical trials related to COVID-19 in order capture rapidly emerging scientific evidence on clinical trial results and new interventions in a timely manner. If relevant scientific evidence emerges on new therapies, the global trial tracker will allow for rapid assessment to allow for new therapies to be added into the TOGETHER Consortium in a timely manner.

In the TOGETHER Consortium, an independent DSMB as well as the international investigators will continuously review the release of data on COVID-19 treatment interventions that can be delivered in outpatient setting. At the discretion of the independent DSMB, interim assessment of interventions may be triggered at any time or during the monthly meetings to assess safety and futility. Interim futility assessment will be done using Cytel's Bayesian predictive power method where a posterior predictive probability of rejecting the null hypothesis at the final analysis will be assessed given the interim data observed at analysis. Data demonstrating futility will lead to the discontinuation of treatment arms with the replacement of other investigational arms. The data from the 3 countries will be combined to rapidly provide efficacy and safety data of interventions and complete trials from each country will not necessarily be required.

In order to detect important treatment effects that are generalizable to subgroups or the larger population, we require large sample sizes representing the subgroups and different geographies. A strategy to overcome the generalizability challenges of individual clinical trials is to test the treatments among different populations in different geographies. In the TOGETHER 1 (US), 2 (Brazil) trials, each setting will test the efficacy, safety and utility of lopinavir-ritonavir in the different geographies taking into account the broad populations as well as subgroups that include sex, disease status, age, co-morbid conditions, race, etc. This will allow the combined analysis to determine whether this intervention can be provided to the broad populations or specific subgroups, as opposed to only detecting effects among specific subgroups in one geographic location, that may indicate type 2 error.

Reason

In response to the CTC discussion and written recommendation dated 27 July 2020 to communicate our study plans and rationale.

Additionally we report that at the TOGETHER DSMB on 23 July 2020, the decision was made to remove HCQ from TOGETHER 1 (US) based on the interim review in favor of lopinavir-ritonavir.

Table of Contents

1. Synopsis 9

2. Schedule of Activities 14

2.1. Schedule of activities for Brazil 14

3. Introduction 16

3.1. Background 16

3.2. Study Rationale 17

3.2.1. COVID-19 and Antiviral Approaches 17

3.2.2. Antiviral Effects of Chloroquine Analogues Against COVID-19 (TOGETHER 1 & 2) 18

3.2.3. Antiviral Effects of Azithromycin Against COVID-19 18

3.2.4. Antiviral Effects of Lopinavir and Ritonavir Analogues Against COVID-19 19

3.2.5. Rationale for Ascorbic Acid Control as a Comparator 19

3.2.6. Rationale for Dosing Selection of Experimental Interventions 19

3.3. Benefit/Risk Assessment 20

4. Objectives and Endpoints 21

4.1. Objectives and Endpoints for Brazil 21

5. Study Design 23

5.1. Overall Design 23

5.1.1. Study Execution for Brazil 24

5.2. Participant and Study Completion 24

6. Study Population 26

6.1. Eligibility criteria for Brazil 26

6.1.1. Inclusion Criteria for Brazil 26

6.1.2. Exclusion Criteria for Brazil 26

6.2. Screen Failures 28

6.3. Recruitment 28

6.4. Co-enrollment Guidelines 28

7. Treatments ... 29
7.1. Treatments Administered ... 29
7.2. Risks to the Participants ... 29
7.2.1. Risks Associated Administration with experimental therapies ... 29
7.2.2. Risks Associated with COVID-19 Diagnosis ... 29
7.2.3. Management of Participants to Limit Risks of SARS-CoV-2 Transmission ... 29
7.3. Dose Modification and Toxicity Management ... 30
7.4. Method of Treatment Assignment ... 30
7.5. Blinding ... 31
7.6. Preparation/Handling/Storage/Accountability ... 31
7.7. Treatment Compliance ... 31
7.8. Concomitant Therapy ... 31
7.9. Treatment After the End of the Study ... 32
8. Discontinuation/Withdrawal Criteria ... 33
8.1. Discontinuation of Study Treatment ... 33
8.2. Withdrawal from the Study ... 35
8.3. Lost to Follow-up ... 36
9. Study Encounters ... 37
9.1. For Brazil ... 37
9.1.1. Screening/Baseline Evaluation: Day minus 1/0 ... 37
9.1.2. Day 1 Through Day 14 ... 38
9.1.3. Day 28 (+/- 5 days) ... 39
9.1.4. Day 56 (+/- 5 days) ... 39
9.1.5. Day 90 or early termination (+/- 5 days) ... 39
9.1.6. Screening/Baseline Evaluation: Day 0/1 ... 39
9.1.7. Day 2 Through Day 13 ... 40
9.1.8. Days 3, 8 and 14 (+/-1 day) ... 40
9.1.9. Day 14 ... 41

9.1.10. Day 28, 56 and 90 41
9.1.11. Participant Reimbursement 41
10. Study Assessments and Procedures 42
10.1. Efficacy Assessments for Brazil 42
10.1.1. Mid-nasal swab or saliva for Brazil 42
10.1.2. Participant Survey for Brazil 42
10.1.3. Patient reported outcomes 43
10.2. Adverse Events for Brazil 43
10.2.1. Definition of Adverse Events for Brazil 43
10.2.2. Adverse Event Reporting for Brazil 44
10.2.3. Assessment of Adverse Events for Brazil 45
10.2.4. Serious Adverse Events for Brazil 46
10.2.5. Treatment of Overdose for Brazil 46
10.2.6. Safety Assessments for Brazil 46
11. Statistical Considerations 46
11.1. Sample Size Determination for Brazil 46
11.2. Populations for Analyses 47
11.3. Statistical Analyses 47
11.3.1. Efficacy Analyses 48
11.3.2. Secondary endpoints 49
11.3.3. Pharmacokinetic Analysis 49
11.3.4. Exploratory Exposure-Response Analyses 49
11.3.5. Combined Study Analysis 50
12. Reimbursement 51
12.1. Reimbursement in Brazil 51
13. References 52

1. Synopsis

Expanded Title:

TOGETHER Trial: An Adaptive Randomized Platform Trial to Investigate the Efficacy of Novel Agents for Treatment of SARS-CoV-2 Infection Among High-Risk Outpatient Adults

Short Title:
Outpatient Management of High-Risk Adults with SARS-CoV-2

Rationale:
Despite the fact that most hospitals are already overwhelmed and hospitalization will not be feasible even for severe COVID-19 cases in most of low- and middle-income countries (LMICs), the majority of current clinical trials is taking place in hospital settings and in high-income countries (HICs) (www.covid19-trials.org). Many of these trials are also excluding marginalized population groups such as people living with HIV (PLHIV) and people with active or latent tuberculosis (TB) who are key populations of interest for LMICs. The burden of HIV and TB in LMICs is generally much higher than in HICs.

There is a need to immediately start the clinical investigation of outpatient strategies in these resource-limited regions that are inclusive of these marginalized groups.

This protocol is for an adaptive platform trial for the treatment of high-risk adults with severe acute respiratory syndrome coronavirus 2 (SARS-CoV-2) infection not requiring hospital admission for Brazil, hereafter referred to as TOGETHER 2. This proposal builds on an US-based adaptive platform trial for high-risk SARS-CoV-2 adult outpatients (hereafter referred to as TOGETHER 1).

The primary research question is to the efficacy of experimental interventions to prevent LRTI among persons with SARS-CoV2 infection who are at high risk of progression. The primary outcomes of trial will include progression to LRTI, defined by SpO_2<93% or 6% decrease in $Sp0_2$ from baseline; and presence of viral shedding from nasal swabs at day 10 of treatment. Other agents are rapidly being screened and developed for SARS-CoV-2 infection and could be incorporated into this protocol as additional arms. The flexible platform trial design will allow additional agents to be added and tested with standardized eligibility criteria, outcomes, and measurements. If an intervention is shown to be effective, this design would allow replacement of the placebo group with the effective intervention as the comparator.

Design:
This is an international multi-center adaptive randomized platform trial for the treatment of SARS-CoV-2 infection in high-risk adults not requiring hospital admission. In Brazil, stratified randomization will be done based on time since symptom onset (<120 hours vs. ≥120 hours) and age (<50 years vs ≥50 years).

Other chloroquine-based therapies, antiviral therapies, and other affordable candidate drug regimens that can be repurposed for COVID-19 may also be considered for this trial. The decision to add new therapeutic strategies will be made based on external findings with consultations of the local stakeholders. Therapies that can be repurposed for COVID-19 (e.g. lopinavir-ritonavir) will be prioritized since they offer more affordable and scalable therapeutic options for LMICs. The added arm may be tested with standardized eligibility criteria, outcomes, and measurements, as the other experimental interventions.

Population:

**Eligibility criteria**
**Brazil**
Men and women 18 years of age to 80 with a clinical condition compatible with COVID-19 and respiratory symptoms:<br>• Persistent dry cough associated with axillary temperature> 37.7o Celsius; or<br>• Recent onset of influenza associated with dry cough; or<br>• Tomographic image compatible with COVID-19 infection<br>Eligible participants will be at increased risk of developing LRTI based on established risk factors for severe COVID-19 disease (at least one of the following):<br>1. Age ≥50 years<br>2. Presence of pulmonary disease, specifically moderate or severe persistent asthma, chronic obstructive pulmonary disease (COPD), pulmonary hypertension, emphysema, or tuberculosis<br>3. Diabetes mellitus (type 1 or type 2), requiring oral medication or insulin for treatment<br>4. Hypertension, requiring at least 1 oral medication for treatment<br>5. Known cardiovascular diseases (CHF of any etiology, documented Coronary Artery Disease, Miscellaneous heart disease with clinical repercussion)<br>6. Symptomatic lung disease and / or being treated<br>7. Patients with a history of transplantation<br>8. Patient with stage IV chronic kidney disease or on dialysis.<br>9. Body mass index ≥30 (self-reported)<br>10. Immunocompromised status due to disease (e.g., those living with human immunodeficiency virus with a CD4 T-cell count of <200/mm^3, confirmed malignancy)<br>11. Immunocompromised status due to medication (e.g., persons taking 20 mg or more of prednisone equivalents a day, anti-inflammatory monoclonal antibody therapies, or cancer therapies)
**Brazil**
• **Lopinavir/ritonavir (LPV/r)**: LPV/r 800 mg-200 mg orally twice daily x 1 day, then 400 mg-100 mg twice daily for an additional 9 days<br>• **Hydroxychloroquine (HCQ)**: 800mg HCQ per day (2 HCQ tablets) for the first day followed by 400mg per day an additional 9 days (1 HCQ tablet)<br>• **Placebo**: 2 placebo pills per day for 10 days
**Brazil**
For evaluations of experimental interventions compared to control, a total of 492 persons per arm will be enrolled.<br>**A second cohort of low-risk participants will not be enrolled in Brazil.**

**Participant counselling and plans for new arms: Brazil**
Participants will be counselled about the preliminary *in vitro* data of the activity of interventions against SARS-CoV-2 and equipoise regarding efficacy in humans given that there are only limited data at this time.<br>If additional data emerge on alternative potentially effective agents for SARS-CoV-2, additional arms can be added to the study as a new intervention appendix to the protocol. The standardized operating procedures and comprehensive statistical analysis plans will allow for integration of new arms.

Objectives and Endpoints:

**Brazil**	
**Objectives**	**Endpoints**
Primary	
• To test whether any of the experimental interventions has an effect on hospitalization and mortality among persons who become hospitalized with COVID-19 disease	• Proportion hospitalized or clinical worsening (in case hospitalization not performed due to lack of hospital beds) for COVID-19 or clinical worsening<br>• Mortality due to complications from COVID-19 after randomization up to 90 days
Secondary	
• To test the efficacy of experimental interventions to prevent progression to LRTI, among persons with SARS-CoV-2 infection who are at high risk of progression<br>• To test the efficacy of experimental interventions to reduce SARS-CoV-2 viral shedding	• LRTI, defined by SpO_2<93% or decline from baseline of 6% in 2 measurements at least 2 hours apart. *Initially, the trial is statistically powered for this endpoint in high-risk population.*<br>• Proportion of persons with clearance of SARS-CoV-2 from nasal swabs or saliva, defined as 1 negative swab.
• To test the safety and adherence of experimental interventions for treatment of high-risk outpatients with SARS-CoV-2 infection	• Serious adverse events (including death, hospitalization) and adverse events resulting in treatment discontinuation up to 28 days

	• Proportion of non-adherent patients with interventions
• To test whether any of the experimental interventions has an effect on hospitalization and mortality among persons who become hospitalized with COVID-19 disease (continued from primary outcome)	• Proportion hospitalized for COVID-19 progression<br>• Days of hospitalization<br>• Mortality for any causes after randomization up to 90 days
• To test whether any of the experimental interventions decrease resolution rate for symptomatic SARS-CoV-2 infection / COVID-19 disease	• Proportion of days with fever after randomization<br>• Proportion of days with respiratory symptoms after randomization<br>• Time until clinical improvement (up to 28 days), defined normalization of temperature, respiratory rate, SaO2, and cough relief (> 50 in relation to baseline measured on a visual analog scale) in the last 72 hours;<br>• Reduction in the perception of dyspnea (upper respiratory tract respiratory symptoms scale - WURSS-11) on days 0, 3, 7, 14 and 28 days;<br>• Time to clinical failure, defined as time to need for hospitalization due to dyspnea, death, need for mechanical ventilation, shock with need for vasoactive amines;
• To test whether any of the experimental interventions is associated with decreased viral shedding from self-collected nasal swabs over 14 days: Day 1, Day 10 and Day 14 (+/-1 days)	• Proportion of days with SARS-CoV-2 detected from mid-nasal swabs by PCR<br>• Median quantity of SARS-CoV-2 detected from mid-nasal swabs by PCR<br>• Change in viral load on day 10 and day 14 after randomization from baseline
• To test the quality-of-life (QoL) of experimental interventions for treatment of high-risk outpatients with SARS-CoV-2 infection	• Change in quality of life measured by Eq-5D-5L from baseline

Exploratory (optional)	
• To assess pharmacokinetics of experimental interventions	• Blood concentration of each experimental intervention in DBS

Sample size:
In Brazil, 492 patients per arm for placebo, HCQ, and LPV/r to achieve 90% power with 0.05 two-sided Type 1 error for a pairwise comparison against the control (ascorbic acid) to detect minimum treatment efficacy defined by 27.5 % relative risk reduction (RRR) of preventing hospitalization assuming a control event rate (CER) of 20%. Eligible participants will be randomized at an equal allocation ratio to study experimental interventions or placebo. In Brazil, if other eligible patient(s) are in the same household, they may be assigned to different regimen.

Duration:
90 days of clinical follow-up per participant
Enrolment and completion TBD based on the observed recruitment rate

Proposed sites: Concurrent with the US site (TOGETHER 1), Brazil (TOGETHER 2), we will work together to generative comparative data. Interventions for TOGETHER 1, 2 and 3 may differ depending on feasibility, evolving epidemiology and the evidence on interventions, and appropriateness of interventions in the local environments.

2. Schedule of Activities

2.1. *Schedule of activities for Brazil*

FLOWCHART	STUDY VISIT SCHEDULE										
	Screening and treatment period							Post-treatment period			
	Screening (1) Visit (D-minus 1)	Baseline and randomization (1) D-0	D1-2 (2) +1 day	D-3(2) +1 day	D4-8(2) +1 day	D-9(2) ±1 day	D-10(2) ±1 day	D-14(2) ±1 day	D-28(2) ±2 day	D-56(2) ±5 day	D-90 or Early Termination (2) ±5 days
Informed Consent	X										
Eligibility Criteria Review	X										
Demography	X										
Medical History	X										
Physical Examination	X										
Temperature	X										
Puloximeter measurement (Pulse rate, HR)		X		X	X (D7)		X	X			
Kardiamobile monitors (QT and heart rate measurement)		X		X	X (D7)		X	X			
Pregnancy Test	X(3)										
Mid-nasal swab or saliva for SARS-COV-2 confirmation and viral load assessment (D0, and D14)	X							X			
Mid-nasal swab or saliva for biobanking deposit (D3, D7 and D10)				X (D3)	X (D7)		X				
Concomitant medications	X			X (D3)	X (D7)		X				
Adverse Events (AEs)		X(4)	X	X	X	X	X	X	X		
Dyspnea Scale (WURSS-11)		X(5)	X	X(5)	X(5)	X(5)	X(5)	X(5)			
Tisdale scale		X									
EQ-5D-5L		X(6)							X(6)		X(6)
Randomization		X(7)									
Administration Investigative treatment (8)		X(9)	X(10)	X(10)	X(10)	X(10)	X(10)				
Medication adherence							X(11) D10				
Verification of clinical outcomes (hospitalization and symptoms)		X(12)	X (13)	X (13)	X (13)	X (13)	X (13)	X (13)	X (13)	X (13)	X (13)
Patient Identification Card		X									

1. Baseline visit may be performed at the same time as the screening visit. For patients without RT-PCR results, quick test for COVID-19 at the screening visit and randomization will be done after confirmation of SARS-CoV-2 infection. For patients with known RT-PCR positive results, screening and baseline visit will be done on the same day.
2. Visits made through telephone contact, calculated in relation to the randomization date. Home visits will be made for mid-nasal or saliva collection.

3. Only women of childbearing potential and / or potential to become pregnant. Urine pregnancy test for women with at least one menstruation in the last 12 months
4. After signing the Informed Consent Form.
5. Scales and questionnaires must be completed BEFORE any procedures of the proposed visit and must be self-applied. Only a person not related to the research can help the patient during the filling. In telephone visits, the patient must respond directly, at the time of contact.
6. Remind the patient that they will answer the questionnaire.
7. After completing the screening / baseline visit procedures and presenting all inclusion / exclusion criteria, patients should be immediately randomized.
8. The study medication will be administered as prescribed.
9. The first treatment dose under investigation must be administered on the same day of randomization (immediately after randomizing)
10. Maintain the administration of the product under investigation according to schedule. Patients administration to study drug as per protocol assessed. Discontinuation if adverse events prevent the medication from continuing.
11. Medication adherence will be assessed on Day 10 over the phone or in person.
12. As soon as the patient start the product under investigation.
13. Verification of clinical outcomes (e.g. symptoms, hospitalization and etc) will be done via telephone. On days when home visits are made, verifications of clinical outcomes may be done in person.

3. Introduction

This is an international multi-center adaptive randomized platform trial for the treatment of severe acute respiratory syndrome coronavirus 2 (SARS-CoV-2) infection in high risk adults not requiring hospital admission. This protocol builds upon the US-based outpatient treatment platform trial.

The trial will initial start with placebo-equivalent (ascorbic acid) as a control in the clinical evaluation of the experimental interventions. Additional arms will be added as new agents or combinations are prioritized. In addition, if one of the agents is found to be superior over placebo, it may become the control group against which new interventions are measured. Evaluations include safety and tolerability, SARS-CoV-2 viral shedding, and development of lower respiratory tract infection (LRTI).

Initially, 165 patients per arm will be recruited and randomized at an equal allocation. The control data (ascorbic acid) from the US trial may be used to in conjunction with the control data from TOGETHER 2 and 3.

In Brazil for TOGETHER 2, the trial will start with three arms:

- 2A) Placebo;
- 2B) HCQ; and
- 2C) LPV/r

During the study participants will perform the following:

- Collect mid-nasal swabs for viral detection for the co-primary trial endpoint
- Complete daily assessments for symptoms of LRTI and measurement of temperature, respiratory rate, pulse, and SpO_2.
- Complete surveys that will include questions about symptoms from both the drug regimen and respiratory and systemic symptoms, review of concomitant medications, and other pertinent topics

During the first 14 study days of follow-up, participants take medication, complete surveys, and collect mid-nasal swab for viral quantification and will assess symptoms for progression to LRTI. Physical assessments will include daily temperature, SpO_2 assessment using a pulse oximeter. The participants will be followed-up to complete surveys on Day 28, Day 56, and Day 90.

3.1. *Background*

SARS-CoV-2 is a coronavirus novel to the human population discovered in December 2019; it is currently the cause of a global pandemic.[1-3] The World Health Organization (WHO) named the novel coronavirus SARS-CoV-2 and the disease caused by SARS-CoV-2 COVID-19. Person-to-person transmission has occurred in China, across temperate Asia, Europe, and North America, with sporadic cases in Africa and person-to-person transmission in the southern hemisphere. Accurate reporting is limited by availability of diagnostic testing. The WHO declared the COVID-19 pandemic a Public Health Emergency of International Concern on 30 January 2020.[4]

Most deaths and severe pneumonitis have occurred in the elderly or in persons with underlying pulmonary or cardiac comorbidities or diabetes. In healthy adults, including pregnant women, it can cause a febrile, self-limited pneumonia. Infection appears less symptomatic in children and

younger adults.[5] Nevertheless, the burden of this pandemic to the global health and economic systems is expected to be substantial. No acquired immunity to this novel viral infection appears to exist in the human population globally, and no effective treatment or preventative agent is licensed at this time.

As with many infectious epidemics, household contacts, first responders, caregivers, and medical personnel attending persons with COVID-19 are at high risk of infection. The incubation time requires 14 days of quarantine for exposed individuals not wearing personal protective equipment [6], and on 03 March 2020, WHO declared a global shortage of personal protective equipment leaving doctors, nurses, and other frontline workers dangerously ill-equipped to care for COVID-19 patients.[7] Extensive absences from the care network and health system will degrade the ability to care not only for those with COVID-19 but also for routine healthcare issues as well. At the height of local epidemic, the health care system becomes overburdened with patients with respiratory illness. To date, rigorous self-isolation and lockdown have been required to contain the SARS-CoV-2, leaving entire societies to abruptly stop normal life. Interventions are urgently needed to stop viral spread and to decrease the morbidity and mortality cause by the infection. The ability to stop viral replication to prevent transmission of the virus and to prevent LRTI, which is associated with need for hospitalization and possibly mechanical ventilatory support, will be of benefit to the individual, the hospital system, and the health of the public. In addition, targeting those at highest risk of progression to LRTI and hospitalization will have the greatest impact on the pandemic. Including a cohort without risk factors for LRTI for the co-primary virologic outcome will provide additional data to inform whether the intervention is likely to have a public health benefit by reducing transmission in situations where self-isolation is not feasible.

3.2. *Study Rationale*

3.2.1. *COVID-19 and Antiviral Approaches*

SARS-CoV-2 is a novel betacoronavirus of zoonotic origin, similar to the coronaviruses severe acute respiratory syndrome coronavirus (SARS-CoV) and Middle East respiratory syndrome coronavirus (MERS). Based on current evidence, case fatality rate for SARS-CoV-2 is about 3%, which is significantly lower than SARS-CoV (10%) and MERS-CoV (40%).[8] However, SARS-CoV-2 has potentially higher transmissibility (R0: 1.4-5.5) than both SARS-CoV (R0: 2-5) and MERS-CoV (R0: <1).[8]

Our understanding of the viral pathogenesis of SARS-CoV-2 remains limited. However, it appears that the virus cell entry depends on binding of the viral spike (S) proteins to cellular receptors and on S protein priming by host cell proteases. SARS-CoV-2, like SARS-CoV, uses the same receptor angiotensin converting enzyme 2 (ACE2) on pulmonary epithelial cells for entry and the transmembrane serine protease 2 for S protein priming.[9] The receptor binding domain of lineage B betacoronaviruses is a single, continuous domain that contains all of the structural information necessary to interact with the host receptor. Fusion is mediated at the cell membrane, delivering the viral nucleocapsid inside the cell for subsequent replication. ACE2 expression is found in the lung epithelial cells, vascular endothelium, renal tubular epithelium, and epithelia of the small intestine. Viral shedding has been localized primarily to respiratory droplets and fecal samples.[2]

Medications to treat and/or prevent SARS-CoV-2 need to inhibit aspects of the viral life cycle, ultimately blocking replication. Already-approved and available medications are ideal for immediate evaluation for SARS-CoV-2 infection treatment and prevention. Two potential targets for anti-SARS-CoV-2 medications are viral polymerases and proteases.[10] Pilot clinical studies are already ongoing for SARS-CoV-2 using various repurposed antiviral medicines (https://www.who.int/emergencies/diseases/novel-coronavirus-2019/global-research-on-novel-

coronavirus-2019-ncov). Similarities between SARS-CoV-2 with SARS-CoV and MERS suggest that antivirals with *in vitro* efficacy against SARS-CoV and MERS may be promising agents as SARS-CoV-2 PEP.[10]

3.2.2. *Antiviral Effects of Chloroquine Analogues Against COVID-19* *(TOGETHER 1 & 2)*

TOGETHER 1 and TOGETHER 2 are studying hydroxychloroquine (HCQ). Chloroquine (CQ) was discovered in 1934 by Bayer and was used in 1945 as an antimalarial to become one of the most prescribed drugs globally, prior to the emergence of widespread drug resistance in *Plasmodium falciparum*.[11] CQ was found to be effective against rheumatoid tenosynovitis in 1951.[12] HCQ was licensed in the United States in 1955 as an antimalarial and as a drug for rheumatoid arthritis, and it was widely marketed for the latter due to a favorable safety profile with chronic use.[13] The mechanisms of action for HCQ for treatment of rheumatoid arthritis and other autoimmune diseases are still not fully understood despite widespread use over the past 60 years.[14]

CQ and HCQ have been proposed as potential agents for treatment and prevention against other infectious agents beyond malaria.[15,16] The mechanism of action differs according to the pathogen: against intracellular bacteria and fungi by alkalinizing vacuoles containing the microorganisms, restoring the activity of other antibiotics, and against viral replication through alkalization of acidic organelles, namely endosomes, lysosomes, and Golgi vesicles.

CQ is effective *in vitro* against SARS-CoV coronavirus in Vero E6 cells with the EC_{50} ~8μM[17] and had shown evidence of prevention activity in vivo.[18] Hence, these re-purposed drugs were obvious hits for testing against SARS-CoV-2. *In vitro* inhibition in Vero E6 cells against the novel coronavirus, SARS-CoV-2, has been published in recent weeks. Wang et al (2020) showed that the EC_{50} and EC_{90} for CQ in Vero E6 cells is 1.13 μM and 6.90 μM, respectively.[19] Yao et al (2020) showed that the EC_{50} for CQ treatment of infected cells at 48 hours was 5.47 μM, whereas HCQ appeared slightly more potent, with EC_{50} of 0.72 μM at 48 hours.[20] These levels appear to be within the range of exposures that could be achieved with standard HCQ treatment, and likely prophylaxis, due to concentrations of the drug achieved in the lung tissue.[20] No *in vitro* data in the lung epithelial cells are available nor are any animal model data.
Multiple observational and small Investigator-initiated COVID-19 pneumonia treatment trials using CQ, HCQ, and variety of other medications are ongoing in China (https://www.who.int/emergencies/diseases/novel-coronavirus-2019/global-research-on-novel-coronavirus-2019-ncov). Gao et al reported anecdotal efficacy of CQ as treatment for COVID-19–associated pneumonia.[21]
Antivirals of relevance to TOGETHER 3:

3.2.3. *Antiviral Effects of Azithromycin Against COVID-19*

Azithromycin is a broad-spectrum azalide antibiotic used to treat a number of bacterial infections, including pneumonia. Azithromycin has shown antiviral activity *in vitro* against Zika, Ebola, rhinoviruses, and other respiratory viruses.[22,23] Although the mechanism of its antiviral activity is not clear, some findings suggest it may be associated with augmentation of interferon response.[23] Alternatively, azithromycin may convey antiviral activity by increasing the pH of cell organelles such as endosomes and the *trans*-Golgi network.[24] Changing the pH of intracellular vesicles may alter the glycosylation of ACE2, a key receptor for cell entry for SARS-CoV-2.[25,26] PK modelling supports the azithromycin regimen will include an additional loading dose on day 1 to allow earlier achievement of azithromycin concentrations in lung that consistently achieve the EC_{50} determined for SARS-CoV-2.

3.2.4. *Antiviral Effects of Lopinavir and Ritonavir Analogues Against COVID-19*

Lopinavir-ritonavir (LPV/r), is a combination medication that has been used to treat HIV/AIDS. Lopinavir is a HIV type 1 aspartate protease inhibitor that is usually “boosted” with ritonavir to increase the plasma half-life of lopinavir through the inhibition of cytochrome P450. Lopinavir has been shown to have in-vitro inhibitory activity against SARS-CoV [**Error! Reference source not found.**, **Error! Reference source not found.**, **Error! Reference source not found.**]. Compared to ribavirin as a historical control group, it has been previously shown that lopinavir–ritonavir (400 mg and 100 mg, respectively) can reduce the risks of adverse clinical outcomes such as acute respiratory distress syndrome and death as well as viral load among patients with SARS. [**Error! Reference source not found.**, **Error! Reference source not found.**, **Error! Reference source not found.**] Based on *in vitro* testing and previous clinical evaluation, LPV/r is an important treatment regimen that should be tested for SARS-CoV-2. Although a recent open-label clinical trial did not show efficacy of LPV/r to decrease days of hospitalization or viral shedding, this trial focused on patients who were already hospitalized, likely due to LRTI and used a suboptimal dosing strategy. The proposed trial will treat participants earlier in the illness, and supported by PK modelling, will use a higher loading doses on day 1, which will allow earlier achievement of LPV concentrations exceeding SARS-CoV-2 EC_{50} values in the lung.

3.2.5. *Rationale for Ascorbic Acid Control as a Comparator*

In healthy adults, COVID-19 disease is likely to present as an upper respiratory viral infection, characterized by a febrile disease with cough and fatigue.[30] Symptom reporting may vary based on participants’ perception as to whether they are taking investigational antivirals or ascorbic acid, but the primary study endpoints of LRTI and viral shedding are not affected. There is no rigorously-proven therapy for individuals with outpatient COVID-19 disease, although multiple therapies are under investigation.

Because there is not established therapy, use of a control is acceptable and ethical both for participants’ health and safety as well as ensuring the most rigorous trial design to evaluate an intervention for COVID-19 disease caused by SARS-CoV-2. As there are multiple intervention regimens with different dosing schedule and route, full blinding for patients and clinicians will not be feasible. Participants will be blinded to their allocation to the extent possible.

The dose of ascorbic acid chosen for this protocol is considered to be safe and well tolerated. All participants, regardless of assigned group, will be able to take additional ascorbic acid (e.g., over the counter vitamins, or through food) should they choose, as there is no known maximum daily safe dose of ascorbic acid. Clinical trial evidence has demonstrated that ascorbic acid, alone or in combination with other micronutrients, does not substantially reduce the risk of upper respiratory infections or severe consequences of infectious processes; thus, ascorbic acid is not expected to have a prevention effect for SARS-CoV-19 and is considered a placebo-equivalent product for this study.

3.2.6. *Rationale for Dosing Selection of Experimental Interventions*

As the COVID-19 epidemic remains very fluid and new data are emerging from observational and clinical trials daily, this protocol is written to allow adaptation to incorporate additional medications throughout the trial. The rationale for dosing selection of each experimental interventions will be provided in each of the corresponding appendix. This modular structure of protocol is used for this platform trial that may end up adding new experimental interventions during the trial.

3.3. *Benefit/Risk Assessment*

There is equipoise as to whether the *in vitro* efficacy of any drug will translate into efficacy to prevent LRTI. COVID-19 disease can be unpredictable in its severity, but a 3.4% mortality rate has been observed among clinical pneumonia cases. The elderly (>60 years) and those with medical comorbidities are at highest risk of poor outcomes.[1-3] Moreover, transmission in younger persons amplifies infection in communities, putting susceptible persons at risk. There is no proven drug for treatment of those with COVID-19 disease.

QT prolongation: Azithromycin and LPV/r are widely used throughout Brazil without the requirement of QT measurements prior to initiation. LPV/r has been reviewed by CredibleMeds (crediblemeds.org) but the evidence available at this time did not result in a decision for it to be placed as a risk medicine for QT prolongation and Torsades de Pointes. Azithromycin has been associated with QT prolongation. However we will exclude patients with a baseline QTc interval of > 470 ms in males, and > 480 ms in females.

LPVr use in undiagnosed HIV-positive patients: Ten days of LPV/r monotherapy is unlikely to cause resistance (high barrier to resistance) and is effective for viral suppression. The risk of HIV-associated immune reconstitution inflammatory syndrome (IRIS) is possible, and the time of onset varies from days to months after antiretroviral initiation. Ten days of LPV/r potentially carries a low risk of unmasking IRIS in undiagnosed HIV-positive patients. Should IRIS occur, it will be recorded as a serious adverse event (SAE). Testing for HIV prior to enrolment is not pragmatic as it exposes health care workers to SARS-CoV-2 infection and there is no referral pathway for newly diagnosed HIV-positive patients with SARS-CoV-2 co-infection. In this study we minimise interaction between study staff and patients using Telehealth.

Drug interactions between study arms and rifampicin-treated and ART treated patients: We included an explicit exclusion criteria to exclude patients with potentially clinically significant pharmacokinetic and pharmacodynamic drug interactions as determined by the study clinical pharmacologist. As far as possible, we want to study HIV-positive and patients treated for pulmonary tuberculosis as they are potentially a high risk for severe disease who could benefit from an early treatment. If one of the study experimental drugs are considered to put the patient at increased risk due to drug interactions, randomization for such patients will be limited to the study arms that are deemed to be safe by the study clinical pharmacologist.

Misclassification as low-risk: We thoroughly question participants about their medical history to best as possible correctly classify them as high- or low-risk for co-morbidities. We will also request participants for consent to access their medical records to review for co-morbidities.

4. Objectives and Endpoints

4.1. *Objectives and Endpoints for Brazil*

Brazil	
**Objectives**	**Endpoints**
Primary	
• To test whether any of the experimental interventions has an effect on hospitalization and mortality among persons who become hospitalized with COVID-19 disease	• Proportion hospitalized or clinical worsening (in case hospitalization not performed due to lack of hospital beds) for COVID-19<br>• Mortality due to complications from COVID-19 after randomization up to 90 days
Secondary	
• To test the efficacy of experimental interventions to prevent progression to LRTI, among persons with SARS-CoV-2 infection who are at high risk of progression<br>• To test the efficacy of experimental interventions to reduce SARS-CoV-2 viral shedding	• LRTI, defined by SpO_2<93% or decline from baseline of 6% in 2 measurements at least 2 hours apart. *Initially, the trial is statistically powered for this endpoint in high-risk population.*<br>• Proportion of persons with clearance of SARS-CoV-2 from nasal swabs or saliva, defined as 1 negative swab
• To test the safety and adherence of experimental interventions for treatment of high-risk outpatients with SARS-CoV-2 infection	• Serious adverse events (including death, hospitalization) and adverse events resulting in treatment discontinuation up to 28 days<br>• Proportion of non-adherent patients with interventions
• To test whether any of the experimental interventions has an effect on hospitalization and mortality among persons who become hospitalized with COVID-19 disease (continued from primary outcome)	• Proportion hospitalized for COVID-19 progression<br>• Days of hospitalization<br>• Mortality for any causes after randomization up to 90 days<br>• Mortality due to pulmonary complications<br>• Mortality due to cardiovascular complications

• To test whether any of the experimental interventions decrease resolution rate for symptomatic SARS-CoV-2 infection / COVID-19 disease	• Proportion of days with fever after randomization<br>• Proportion of days with respiratory symptoms after randomization<br>• Time until clinical improvement (up to 28 days), defined normalization of temperature, respiratory rate, SaO2, and cough relief (> 50 in relation to baseline measured on a visual analog scale) in the last 72 hours;<br>• Reduction in the perception of dyspnea (upper respiratory tract respiratory symptoms scale - WURSS-11) on days 0, 3, 7, 14 and 28 days;<br>• Time to clinical failure, defined as time to need for hospitalization due to dyspnea, death, need for mechanical ventilation, shock with need for vasoactive amines;
• To test whether any of the experimental interventions is associated with decreased viral shedding from self-collected nasal swabs over 14 days: Day 1, Day 10 and Day 14 (+/-1 days)	• Proportion of days with SARS-CoV-2 detected from mid-nasal swabs by PCR<br>• Change in viral load on day 10 and day 14 after randomization from baseline
• To test the quality-of-life (QoL) of experimental interventions for treatment of high-risk outpatients with SARS-CoV-2 infection	• Change in quality of life measured by Eq-5D-5L from baseline

5. Study Design

5.1. *Overall Design*

The overarching goal of this study is to assess the effectiveness of interventions on important clinical and viral outcomes among high-risk adult outpatients with SARS-CoV-2 infection to inform public health control strategies.

This is an international multi-center adaptive randomized platform trial for the treatment of SARS-CoV-2 infection in high-risk adults not requiring hospital admission.

Initially, the Brazil study will enrol 492 patients per arm with high-risk for hospitalization and LRTI progression at baseline who are PCR-confirmed SARS-CoV-2 infection.

In Brazil for TOGETHER 2, the trial will start with three arms:

- **Placebo**: Placebo pills acid 1000 mg orally twice daily for 1 day then 500 mg orally twice daily for 9 days
- **Lopinavir/ritonavir (LPV/r)**: LPV/r 800 mg-200 mg orally twice daily x 1 day, then 400 mg-100 mg twice daily for an additional 9 days
- **Hydroxychloroquine (HCQ)**: 800mg HCQ per day (2 HCQ tablets) for the first day followed by 400mg per day an additional 9 days (1 HCQ tablet)

Other chloroquine-based therapies, antiviral therapies, azithromycin, and other affordable candidate drug regimens that can be repurposed for COVID-19 may also be considered for this trial. The decision to add new therapeutic strategies will be made based on external findings with consultations of the local stakeholders. Therapies that can be repurposed for COVID-19 will be prioritized since they offer more affordable and scalable therapeutic options for LMICs. The added arm will be tested with standardized eligibility criteria, outcomes, and measurements, as the other experimental interventions.

An independent data and safety monitoring board (DSMB) will be convened for this study with expertise in COVID-19 or respiratory viruses and emerging epidemics as well as biostatistics. The purpose of the DSMB is to monitor the study for operational futility, social harms, and efficacy. The DSMB will also review the blinded sample size re-assessment plan, proposed sample size changes, and make recommendations on the allocation ratio, in case the new intervention arms are added. If additional data emerge on alternative effective agents, the protocol could be modified through an amendment to alter its sample size and evaluate alternative therapies.

A blinded sample size re-assessment will be done to potentially increase the sample size target during the trial, should a lower control event rate (CER) and/or higher drop-out rate be observed. The decision on the timing of the sample size re-assessment will be made by the independent DSMB during the trial based on the observed recruitment rate. The DSMB will also decide on a possible interim analysis during the trial after the sample size re-assessment based on the information on the observed recruitment rate, the CER, the drop-out rate, and the final sample size target, if applicable.

The decision for interim analysis will be made in a blinded manner (e.g., based on pooled number of events). The interim monitoring plan (written by the Study Statistician) will define monitoring bounds to maintain the two-sided type I error rate at the desired 5% (e.g. 97.5% or higher

probability of superiority over the control group). Should a new experimental candidate be added during the trial, allocation ratios will be adapted to favor the new arm.

If additional data emerge on alternative potentially effective agents for SARS-CoV-2, additional arms can be added to the study as a new intervention appendix to the protocol. The standardized operating procedures and comprehensive statistical analysis plans will allow for integration of new arms.

5.1.1. *Study Execution for Brazil*

This study will be carried in two stages: *Internal Pilot and Main Stages*

Due to the rapid evolution of the COVID-19 pandemic and the challenge that public health systems will face to respond to this devastating infection, there are several aspects related to the feasibility of the study that need to be evaluated as soon as we start implementing it.

The aim of the internal pilot phase is to assess any unpredictable feasibility problems and resolve them to improve the overall success of the research. In particular, we will assess issues related to drug recruitment, consent, availability and administration, data collection and recording. This will involve about 18-20 patients in total. There will be no statistical analysis at the end of the pilot stage. The pilot and main stages will be operationally connected with the patients enrolled in the pilot stage will be transferred to the main stage during which the recruitment efforts will be amplified.

The pilot stage will start with two arms: HCQ and placebo, with LPV/r possibly being added if the drug supply becomes available during the pilot stage.

The main stage will begin with three arms: HCQ, LPV/r, and placebo. Safety profile of the monotherapy arms will be tested after being administered for 100 patients per arm. This will be reviewed internally and externally with AbbVie. If adequate safety profile is observed in both monotherapies, combined arm of HCQ + LPV/r will be added into the TOGETHER 2 with permission from AbbVie.

With the time required to review the safety profile of HCQ and LPV/r monotherapy with AbbVie, (assuming adequate safety profile) the fourth arm of combined HCQ + LPV/r will likely be introduced into the trial after enrolment of 165 per arm has already been randomized to the three initial groups of placebo, HCQ, and LPV/r.

For the clinical evaluation of HCQ + LPV/r arm versus placebo, we will use unequal allocation of 3:1 (165 for combined arm and additional 55 for placebo arm) and will incorporate the previously placebo control data by borrowing the individual treatment effects of HCQ and LPV/r versus placebo as empirical prior for the clinical evaluation of the combined arm.

5.2. *Participant and Study Completion*

As an initial target, up to 165 high risk participants per arm will be randomly assigned to study treatment or control in Brazil. There may be more than 165 patients randomized to the placebo arm during the entire duration of the trial given the possible addition of HCQ+LPV/r arm.

This sample size target will likely increase based on the blinded sample size re-assessment.

A participant is considered to have completed the study if he/she has completed all phases of the study including the last scheduled procedure shown in the Schedule of Activities (SoA). The clinical evaluation of each experimental intervention will be considered completed when sufficient number of participants complete the study to enable appropriate evaluation of the primary endpoint.

6. Study Population

Prospective approval of protocol deviations to recruitment and enrolment criteria, also known as protocol waivers or exemptions, is not permitted.

6.1. *Eligibility criteria for Brazil*

6.1.1. *Inclusion Criteria for Brazil*

- Completed informed consent form
- Men and women 18 years of age or older with persistent dry cough associated with axillary temperature> 37.7o Celsius; or recent onset of influenza associated with dry cough
- Laboratory confirmed SARS-CoV-2 infection, with test results within past 72 hours
- Possesses at least one of the following risk-factors for severe COVID-19 disease
    - Risk factors:
        1. Age ≥50 years
        2. Presence of pulmonary disease, specifically moderate or severe persistent asthma, chronic obstructive pulmonary disease (COPD), pulmonary hypertension, emphysema, or tuberculosis
        3. Diabetes mellitus (type 1 or type 2), requiring oral medication or insulin for treatment
        4. Hypertension, requiring at least 1 oral medication for treatment
        5. Known cardiovascular diseases (CHF of any etiology, documented Coronary Artery Disease, Miscellaneous heart disease with clinical repercussion)
        6. Symptomatic lung disease and / or being treated
        7. Patients with a history of transplantation
        8. Patient with stage IV chronic kidney disease or on dialysis.
        9. Immunocompromised status due to disease (e.g., those living with human immunodeficiency virus with a CD4 T-cell count of <200/mm^3, confirmed malignancy)
        10. Immunocompromised status due to medication (e.g., persons taking 20 mg or more of prednisone equivalents a day, anti-inflammatory monoclonal antibody therapies, or cancer therapies)
        11. Patients with cancer
        12. Body mass index ≥30 (self-reported or measured)

- Eligible patients must be willing to use the proposed investigational treatment and have ability to provide informed consent.

6.1.2. *Exclusion Criteria for Brazil*

Participants are excluded from the study if any of the following criteria apply:

1. RT-PCR exam for SARS-CoV-2 negative during the screening visit
2. Known hypersensitivity to any of the study drugs

3. Patients with an acute respiratory condition and with moderate to high probability of not being a SARS-CoV-2 infection
4. Currently hospitalized
5. Severe respiratory clinical condition, presenting at least ONE of the criteria below:
   - The respiratory rate> 28 / min;
   - SaO2 <90% with nasal oxygen therapy at 10 l / min;
   - PaO2 / FIO2 <300 mmHg
6. History of Cardiac Arrhythmia or Long QT Syndrome;
7. Use of Medications that are known to prolong QTc: Citalopran, Venlaflaxina, Bupropion and with no possibility of suspension during the period of use of medications.
8. Inability to take oral medications;
9. Patients on continuous use of Amiodarone and / or PGE5 Inhibitors (Ex: Sildenafil and similar).
10. Use of Digoxin, Cyclosporine, Cimetidine, Tamoxifen
11. Use of anticonvulsants, antifungals, immunosuppressants other than corticotherapy.
12. Use of Hydroxychloroquine for other indications
13. Use of chemoprophylaxis for malaria.
14. Psoriasis in a form other than cutaneous
15. Porphyria
16. Use of protease inhibitors, ritonavir or Cobicistat
17. Clinical history of Liver Cirrhosis or Child-Pugh C classification;
18. Patients with a history of degenerative retinal diseases (patients with retinal diseases due to diabetes and hypertension may participate in the research);
19. Patient with a clinically relevant history of hearing loss;
20. Patients with known severe degenerative neurological diseases and / or severe mental illness;
21. Inability of the patient or representative to give consent or adhere to the procedures proposed in the protocol;
22. Taking chronic medications associated with prolonged QT and may induce Torsades de Pointes as per CredibleMeds.org, including certain antipsychotic medications or antidepressants (e.g., citalopram, venlafaxine, and bupropion) and unable to stop during the trial
23. Baseline QTc interval of > 470 ms in males, and > 480 ms in females
24. Potentially clinically significant pharmacokinetic and pharmacodynamic drug interactions as determined by the study clinical pharmacologist**
25. Pregnancy or lactating women

Note:

*Patients who have already started on the assigned therapy may stop treatment if they test negative for SARS-COV-2 during screening

**If randomization to one study arm may be considered to put the patient at increased risk due to drug interactions, randomization will limited to the arms with safer interventions. This is important to allow participation of HIV-infected participants on treatment and rifampicin-treated participants.

6.2. *Screen Failures*

Screen failures are defined as participants who consent to participate in the clinical study but are not subsequently randomly assigned to study treatment. A minimal set of screen failure information is required to ensure transparent reporting of screen failure participants to meet the Consolidated Standards of Reporting Trials publishing requirements and to respond to queries from regulatory authorities. Minimal information includes demography, screen failure details, eligibility criteria, and any serious adverse event (SAE).

Individuals who do not meet the criteria for participation in this study (screen failure) can be rescreened if there is a change in their eligibility.

6.3. *Recruitment*

Each site will establish local recruitment and screening methods that operationalize protocol-specified requirements for eligibility determination in a manner that is tailored to and most efficient for the local study setting and target study population. Each site will use a variety of recruitment approaches, including direct recruitment at clinics, referrals from other providers and SARS-CoV-2 testing sites and laboratories, and use of online and social networking websites and apps. Recruitment materials will educate participants about COVID-19, transmission within households, and epidemiology in the community. *See Appendix 5.*

The proposed sites have established track records of high-quality clinical research integrated into clinical care settings; annual retention rates in clinical trials conducted in these sites exceed 90%. The sites have large COVID-19 epidemics with regulation limiting contact to reduce infectious spread.

6.4. *Co-enrollment Guidelines*

Participants may be co-enrolled in other research studies, provided that these are observational studies only. Any other exception requires approval of the Principal Investigators; if a participant clinically worsens, such as requiring hospitalization, it is expected that an exception will be automatically granted and participation in treatment studies permitted. The study team should be consulted for co-enrollment in studies that do not meet this guidance or if there are questions about eligibility for co-enrollment. For any co-enrolled study, combined blood draws should not exceed current Red Cross phlebotomy guidance.

7. Treatments

Study treatment is defined as any investigational treatment(s), marketed product(s), placebo, or medical device(s) intended to be administered to a study participant according to the study protocol.

7.1. *Treatments Administered*

Drug	Dosage formulation	Route of Administration	Manufacturer	Instructions
**Placebo arm for Brazil**				
Placebo pills	--	Oral	The placebo pills will be manufactured by the local certified pharmaceutical labs.	Take 2 tablets pills per day for 10 days

Packaging and label: The medication will be dispensed in an otherwise-unmarked container with the study label. The container will be labeled with a unique identifier.

For the experimental interventions, the details on the dosage formulation, route of administration, manufacturer, and instruction are provided in their corresponding Appendix.

7.2. *Risks to the Participants*

7.2.1. *Risks Associated Administration with experimental therapies*

The safety of experimental therapies for treatment of patients with COVID-19 disease is unknown. However, their side effect profiles are well described, and these drugs are generally well tolerated. This protocol is using a short loading dose, not a sustained one.

COVID-19 may regardless be associated with cardiac effects. The proposed regimens may prolong QT, resulting in arrhythmias. Participants will be screened for QT prolongation. Long-term manifestations of LPV/r and other regimens, including hepatic disease, are not likely in short term exposure.

The risks associated with administration of each experimental intervention is provided in their corresponding appendix.

7.2.2. *Risks Associated with COVID-19 Diagnosis*

Enrollment in this protocol will not impact the public health department's advice for self-quarantine. Enrollment may improve morale during quarantine for COVID-19 infection. COVID-19 may be associated with anxiety, and the ability to monitor for LRTI and interact with study clinicians may allay anxiety.

7.2.3. *Management of Participants to Limit Risks of SARS-CoV-2 Transmission*

To limit the transmission of SARS-CoV-2, participants will receive visits via secure Telehealth in order to limit the movement of persons with potential SARS-CoV-2 and leave clinical space free for ill patients requiring care. Also, to limit exposure in waiting rooms clinical specimens will be

self-collected and collected by a study driver. This will also eliminate exposure of study personnel to SARS-CoV-2.

7.3. *Dose Modification and Toxicity Management*

If a study therapy dose is missed, it should be taken as soon as possible. If it is less than 4 hours before the next dose, the dose should be skipped.

Modification for toxicities is discussed below. Only toxicities related to study medications provided through the study will be considered in the toxicity management section.

Grade 1 or 2

Participants who develop Grade 1 or 2 toxicity (per division of acquired immunodeficiency syndrome [DAIDS] adverse event [AE] Grading Table; see: https://rsc.tech-res.com/docs/default-source/safety/division-of-aids-(daids)-table-for-grading-the-severity-of-adult-and-pediatric-adverse-events-corrected-v-2-1.pdf) that is considered to be related to the study medication may continue study treatment at the discretion of the site Investigator with close follow-up. If a participant chooses to discontinue study treatment, the site should notify the study protocol team within 7 days. These participants will remain on study, off study treatment, and have all evaluations performed.

Grade 3

- Participants who develop a Grade 3 symptomatic toxicity thought by the site Investigator to be related to study drug should have study product withheld, and the site should consult with the Core Protocol team. The participant should be reevaluated every 2 days until the AE returns to Grade ≤2, at which time study drug may be reintroduced at the discretion of the site Investigator in consultation with the protocol team.
- Participants experiencing Grade 3 toxicity requiring permanent discontinuation of study product should be followed up weekly until resolution of the toxicity. Participants will have premature study treatment discontinuation evaluations performed. These participants will remain on study, off study treatment, and have all evaluations performed per the SoA.

Grade 4

- Participants who develop a Grade 4 symptomatic toxicity will have study product permanently discontinued, and the site should notify the Principal Investigator within 72 hours.
- Participants experiencing Grade 4 toxicity requiring permanent discontinuation of study product should be followed up weekly until resolution of the AE or return to baseline. These participants will remain on study, off study treatment, and have all evaluations performed per the SoA.

Specific Management of Toxicities Related to Study-Provided Drugs

Specific management details of toxicities related to study provided drugs are provided in their corresponding appendix.

7.4. *Method of Treatment Assignment*

Participants will be randomized at an equal allocation ratio between experimental intervention and control groups at the level of the individual.

The randomization plan will be overseen by the Study Statistician. The randomization code and resulting allocation list will be generated and overseen by the Study Statistician. The list will be blocked and stratified by site and risk level.

7.5. *Blinding*

As there are multiple intervention regimens with different dosing schedule and route that make blinding for patients and clinicians will not be feasible. The medications are not identical-appearing and dosing is different for the active agents so blinding will operate in different ways across those involved in the study. This study will preserve blinding for the laboratory testing and study statisticians.

- The bottle of medication they receive will not identify the treatment allocation, only the number and frequency of pills to be taken. However, full blinding of patients will likely not be possible.
- While the study medication will be dispensed directly to participants, full blinding of the treating clinicians will not be feasible given that there are multiple intervention regimens.
- Laboratory testing for viral shedding will be blinded, as laboratory staff will not be informed of randomized assignment. The viral shedding endpoint of the trial is an objective one, unlikely to be altered by unmasking, should it occur.
- Study pharmacy staff will be unblinded, as they will prepare the study medication.
- The study statistician will be blinded for analysis purposes.

LPV/r and other regimens have a different dosing schedule and a different shape but study clinicians will not see the study medication, to mimic some blinding.

The participants will be blinded to their randomization group once assigned. At enrollment, the unblinded Study Pharmacist will use the randomization code revealed at the point of randomization to provide the participant with their group assignment and dispense the allocated study medication in a bottle marked with the study label. The medication and medication information, mid-nasal swabs sufficient to complete the study procedures, *DBS sampling kit, if within the sub-study,* and study instructions will be provided to the participant.

7.6. *Preparation/Handling/Storage/Accountability*

Drugs should be stored at room temperature, as per package insert. Records must be maintained that document receipt, release for dosing, disposal, or return to the sponsor.

7.7. *Treatment Compliance*

The participant will be contacted to ensure that they received the box of study supplies; were able to collect the mid-nasal swab and store it appropriately; and took their medication as prescribed. Participants will be asked to complete a survey that includes information regarding treatment administration. *In a sub-study, azithromycin and lopinavir-ritonavir concentrations via a DBS will also be evaluated.*

Consultation via Telehealth, text messaging, or telephone will be available to provide support to the participant to complete study procedures.

7.8. *Concomitant Therapy*

Participants will be asked about concomitant medications at the screening/baseline evaluation visit. During the study, participants will be asked to complete Surveys (Daily Survey and Exit Contact Survey) that include information regarding any medication or vaccine (including

over-the-counter or prescription medicines, vitamins, and/or herbal supplements) that the participant receives during the study. At each contact, the Investigator should question the participant about any medication taken. Participants taking hormonal contraceptives to prevent pregnancy will be educated that they will need to use a backup form of birth control during the study period.

7.9. *Treatment After the End of the Study*

No additional treatment will be provided at the end of the study.

8. Discontinuation/Withdrawal Criteria

8.1. *Discontinuation of Study Treatment*

Study treatment will be discontinued for the following reasons:

- Hospitalization
- Requirement for prohibited concomitant medications or other contraindication to study product
- Occurrence of an AE requiring discontinuation of study product
- Request by participant to terminate study treatment

8.1.1. *Clinical reasons believed to be life-threatening by the physician, even if not addressed in section 7.2.Screening/Baseline Evaluation: Day 0/1*

Screening and enrolment will be done with the participant physically present.

Day 0 evaluations are as follows:

- Informed consent
- Collection of demographic information
- Collection of past and current medical conditions, including known pregnancy and/or lactation status
- Collection of concomitant medication information
- Collection of information regarding exposure to the index case
- Check of inclusion and exclusion criteria
- Collect mid-nasal swab for PCR

Eligible participants will be randomized. Participants will receive a monitoring kit at enrolment, which will include thermometer and SpO_2 device. Participants will be instructed how to self-assess respiratory rate, temperature and oxygen saturation. A study driver wearing personal protective equipment will deliver and collect study related mid-nasal swabs and DBS cards as needed.

The participant will do the following on Day 1:

- Take study therapy (as assigned)
- Complete Daily Survey (online, telephone, or text messaging). This Survey will include confirmation of adherence to study therapy dosing and daily swab collection, concomitant medication review, and symptoms review and objective measures including SpO_2, respiratory rate, temperature and pulse. In addition, the Baseline Dyspnea Index survey will be completed.

Instructions for skin puncture and DBS sample preparation are provided in Appendix 4. A study team member will be available via Telehealth, telephone, or text messaging to provide support for completion of this study procedure.

Screening/Day 0 and Day 1 procedures can occur on the same day.

The SARS-CoV-2 positive test results will be confirmed through laboratory records.

8.1.2. *Day 2 Through Day 13*

The participant will do the following every day from Day 1 through Day 13, inclusive:

- Collect mid-nasal swab for PCR on Day 3, Day 5, Day 7 and Day 10. (Swab collection may be decreased to Day 5 and Day 10 in participants where multiple swabbing is not feasible.)
- Take study therapy (as assigned)

- Complete Daily Survey (online, telephone, or text messaging). This Survey will include confirmation of adherence to study therapy dosing and daily swab collection, concomitant medication review, AEs, and symptoms review and objective measures including SpO_2, respiratory rate, temperature and pulse. In addition, the Transitional Dyspnea Survey will be completed.
- *If in DBS Sub-study, collect DBS samples for analysis of concentration of assigned experimental intervention at any time during this period (1 to 5 times) after study drug dosing has commenced. No more than 1 sample per day should be collected.*

A study team member will be available via Telehealth, text messaging, or telephone to provide support for completion of study procedures. The driver will collect the swabs as required.

8.1.3. *Days 3, 8 and 14 (+/-1 day)*

Contact with study clinician or staff will be conducted via telemedicine (Telehealth) or telephone. Participants will be clinically assessed for signs and symptoms of respiratory distress and will have in person assessment of AEs. As needed, additional contact with the study clinician or staff will be conducted at the request of the participant (e.g., if developing concerning symptoms or an adverse event) or if needed to clarify study procedures or follow-up symptoms.

8.1.4. *Day 14*

The participant will do the following on Day 14:

- Collect mid-nasal swab for PCR (May be omitted in participants where multiple swabbing is not feasible).
- Complete Daily Survey (online, telephone, or text messaging). This Survey will include confirmation of adherence to study therapy dosing and daily swab collection, concomitant medication review, and symptoms review and objective measures including SpO_2, temperature and pulse.
  -
- *If in DBS Sub-study, collect DBS samples for analysis of concentration of assigned experimental intervention, if not already collected.*

A study team member will be available via Telehealth, text messaging, or telephone to provide support for completion of study procedures.
Clinical outcomes will be confirmed through the electronic health record, if possible.

8.1.5. *Day 28, 56 and 90*

Participants will be contacted on days 28, 56 and 90 to determine well-being. On day 56 a DBS may be collected to determine antibody development

8.1.6. *Participant Reimbursement*

Participants will be reimbursed on Day 14. No reimbursement will be provided to index cases for questionnaire completion and referral of their close contacts. No reimbursement will be provided for unscheduled Telehealth visits requested by the participants for support with study procedures.

9. Study Assessments and Procedures

- Study procedures and their timing are summarized in the SoA.
- Protocol waivers or exemptions are not allowed.
- Immediate safety concerns should be discussed with the sponsor immediately upon occurrence or awareness to determine if the participant should continue or discontinue study treatment.
- Adherence to the study design requirements, including those specified in the SoA, is essential and required for study conduct.
- All baseline evaluations must be completed and reviewed to confirm that potential participants meet all eligibility criteria. The Investigator will maintain a screening log to record details of all participants screened and to confirm eligibility or record reasons for screening failure, as applicable.
- Procedures conducted as part of the participant's routine clinical management and obtained before signing of the informed consent form may be utilized for screening or baseline purposes provided the procedures met the protocol-specified criteria and were performed within the time frame defined in the SoA.
- *Blood samples will only be collected as a part of a sub-study. The maximum amount of blood collected from each participant over the duration of the study, including any extra assessments that may be required, will not exceed 10 mL.*
-

Participants who stop study product should continue study participation off study product with continued evaluations as per the SoA. The reason for study product discontinuation should be recorded.

9.1. *Withdrawal from the Study*

- A participant may withdraw from the study at any time at his/her own request or may be withdrawn at any time for the following reasons:
  - At the request of the primary care provider if he/she thinks the study is no longer in the best interest of the participant
  - Participant is judged by the Investigator to be at significant risk of failing to comply with the provisions of the protocol as to cause harm to self or seriously interfere with the validity of the study results
  - At the discretion of the Institutional Review Board/Ethics Committee or government agencies as part of their duties, Investigator, or industry supporter
- If the participant withdraws consent for disclosure of future information, the sponsor may retain and continue to use any data collected before such withdrawal of consent.
- If a participant withdraws from the study, he/she may request destruction of any samples taken and not tested, and the Investigator must document this in the site study records.
- See SoA for data to be collected at the time of study discontinuation and follow-up and for any further evaluations that need to be completed.

9.2. *Lost to Follow-up*

A participant will be considered lost to follow-up if he/she is unable to be contacted by the study site.

The following actions must be taken if a participant fails to comply with required study procedures:

- The site must attempt to contact the participant as soon as possible and counsel the participant on the importance of maintaining the assigned procedure schedule and ascertain whether or not the participant wishes to and/or should continue in the study.
- Before a participant is deemed lost to follow-up, the Investigator or designee must make every effort to regain contact with the participant (where possible, 3 telephone calls and, if necessary, a certified letter to the participant's last known mailing address or local equivalent methods). These contact attempts should be documented in the participant's medical record.
- Should the participant continue to be unreachable, he/she will be considered to have withdrawn from the study with a primary reason of lost to follow-up.

10. Study Encounters

10.1. *For Brazil*

The current COVID-19 pandemic has placed a significant burden on the healthcare system. For this study, specimen and data collection will be conducted to minimize impact of non-ill participants within the healthcare system. If the participant is assessed as eligible, contact between study participants and study personnel will occur in person should the participants are identified at primary care setting, via a telephone, or through home visits by local physician assistants and medical students with PPE.

Participants will be instructed to seek clinical care should they manifest any signs or symptoms of LRTI requiring medical intervention.

10.1.1. *Screening/Baseline Evaluation: Day minus 1/0*

Participants will be assessed for study eligibility through a screening conducted through a home visit. Screening/Day minus 1 and Day 0 procedures can occur on the same day.

Day minus 1 evaluations are as follows:

- Informed consent
- Eligibility criteria review
- Collection of demographic information
- Use of tobacco and alcohol
- Illegal substance abuse
- Collection of past and current medical conditions, including known pregnancy and/or lactation status
- Urine pregnancy test for women with at least one menstruation in the last 12 months
- Nasopharyngeal or saliva sample collection for RT-PCR for SARS-CoV-2 confirmation
- Collection of information regarding exposure to the index case
- Assessment of respiratory signs and symptoms

The participant will do the following on Day 0:

- Interactive Web Response System (IWRS) registration and randomization after PCR confirmation (the expected turn around for PCR testing is 12-24 hours)
- Recheck the inclusion / exclusion criteria and verify the participant's interest in continuing in the study
- Physical examination, including weight, height, vital signs measurement (seated/supine PA, pulse rate, and temperature)
- Collection of concomitant medication information
- Delivery of medications and guidelines for the assigned medications
- Delivery of medicines under investigation. The patient will receive the drugs under investigation as allocated by IWRS (patients in the placebo group will receive the standard treatment defined by the assistant team) and the necessary instructions for use.

- Patient will receive a card with 24-hour telephone contact to call if necessary. Patient will be guided on daily telephone contact and on the collection kits for viral load research.
- Take study therapy (as assigned)
- Tolerance towards the assigned therapy
- Confirmation of adherence to study therapy dosing
- Daily dosing time
- Reporting of adverse events
- Cardiac measurement using Kardiamobile monitors
- SpO_2, respiratory rate, temperature and pulse measurements
- Measurement of heart rate (pulse oximeter) at rest after the participant remained seated at rest for 5 minutes
- Blood pressure measurement after the participant was seated at rest for 5 minutes
- Clinical symptoms review
- WURSS-11 survey for dyspnea
- Tisdale scale
- EQ-5D-5L
- Guidance on daily telephone contacts
- COVID-19 guidelines and quarantine recommendations

Eligible participants will be randomized. Participants will be instructed how to self-assess respiratory rate. Local health care providers wearing personal protective equipment will deliver and collect study related mid-nasal swabs and DBS cards as needed.

Participants will be assessed at the home visits during the mid-nasal swab or saliva collection thermometer, Kardiamobile device, and pulse oximetry device.

The SARS-CoV-2 positive test results will be confirmed through laboratory records.

Patients tested negative for COVID-19 will be considered a screening failure, and only positive patients will be treated and invited for subsequent follow-ups.

10.1.2. *Day 1 Through Day 14*

The participant will do the following every day from Day 2 through Day 14 (unless stated otherwise), inclusive:

- Take study therapy (for a total treatment of 10 days of treatment)
- Tolerance towards the assigned therapy
- Confirmation of adherence to study therapy dosing (Day 10)
- Daily dosing time from the day prior
- Reporting of adverse events
- Cardiac measurement using Kardiamobile monitors (Day 3, Day 7, Day 10, and Day 14)
- Pulseoximeter measurements (Day 3, Day 7, Day 10, and Day 14)

- Measurement of heart rate (pulse oximeter) at rest after the participant remained seated at rest for 5 minutes
- Review of clinical symptoms and hospitalization
- WURSS-11 survey for dyspnea

A study team member will be available via Telehealth, text messaging, or telephone to provide support for completion of study procedures. Participants will be clinically assessed for signs and symptoms of respiratory distress and will have in person assessment of AEs. As needed, additional contact with the study clinician or staff will be conducted at the request of the participant (e.g., if developing concerning symptoms or an adverse event) or if needed to clarify study procedures or follow-up symptoms.

The health care provider will make home-visits to collect nasal-swabs or saliva samples for RT-PCR on Day 3, Day 7, Day 10, and Day 14. These samples collected on Day 14 will be used to analyse the viral shedding related outcomes. Other samples collected on Day 3, Day 7 and Day 10 will be stored for a biobanking deposit.

A study team member will be available via Telehealth, text messaging, or telephone to provide support for completion of study procedures.

Clinical outcomes will be confirmed through the electronic health record, if possible.

10.1.3. *Day 28 (+/- 5 days)*

Participants will be followed-up via Telehealth, text messaging, or telephone:

- EQ-5D-5L
- Review of AEs, clinical symptoms, and hospitalization

10.1.4. *Day 56 (+/- 5 days)*

Participants will be followed-up via Telehealth, text messaging, or telephone:

- Review of clinical symptoms, and hospitalization

10.1.5. *Day 90 or early termination (+/- 5 days)*

Participants will be followed-up via Telehealth, text messaging, or telephone:

- Review of clinical symptoms, and hospitalization
- EQ-5D-5L

10.1.6. *Screening/Baseline Evaluation: Day 0/1*

Screening and enrolment will be done with the participant physically present.

Day 0 evaluations are as follows:

- Informed consent
- Collection of demographic information
- Collection of past and current medical conditions, including known pregnancy and/or lactation status
- Collection of concomitant medication information
- Collection of information regarding exposure to the index case

- Check of inclusion and exclusion criteria
- Collect mid-nasal swab for PCR

Eligible participants will be randomized. Participants will receive a monitoring kit at enrolment, which will include thermometer and SpO_2 device. Participants will be instructed how to self-assess respiratory rate, temperature and oxygen saturation. A study driver wearing personal protective equipment will deliver and collect study related mid-nasal swabs and DBS cards as needed.

The participant will do the following on Day 1:

- Take study therapy (as assigned)
- Complete Daily Survey (online, telephone, or text messaging). This Survey will include confirmation of adherence to study therapy dosing and daily swab collection, concomitant medication review, and symptoms review and objective measures including SpO_2, respiratory rate, temperature and pulse. In addition, the Baseline Dyspnea Index survey will be completed.

Instructions for skin puncture and DBS sample preparation are provided in Appendix 4. A study team member will be available via Telehealth, telephone, or text messaging to provide support for completion of this study procedure.

Screening/Day 0 and Day 1 procedures can occur on the same day.
The SARS-CoV-2 positive test results will be confirmed through laboratory records.

10.1.7. *Day 2 Through Day 13*

The participant will do the following every day from Day 1 through Day 13, inclusive:

- Collect mid-nasal swab for PCR on Day 3, Day 5, Day 7 and Day 10. (Swab collection may be decreased to Day 5 and Day 10 in participants where multiple swabbing is not feasible.)
- Take study therapy (as assigned)
- Complete Daily Survey (online, telephone, or text messaging). This Survey will include confirmation of adherence to study therapy dosing and daily swab collection, concomitant medication review, AEs, and symptoms review and objective measures including SpO_2, respiratory rate, temperature and pulse. In addition, the Transitional Dyspnea Survey will be completed.
- *If in DBS Sub-study, collect DBS samples for analysis of concentration of assigned experimental intervention at any time during this period (1 to 5 times) after study drug dosing has commenced. No more than 1 sample per day should be collected.*

A study team member will be available via Telehealth, text messaging, or telephone to provide support for completion of study procedures. The driver will collect the swabs as required.

10.1.8. *Days 3, 8 and 14 (+/-1 day)*

Contact with study clinician or staff will be conducted via telemedicine (Telehealth) or telephone. Participants will be clinically assessed for signs and symptoms of respiratory distress and will have in person assessment of AEs. As needed, additional contact with the study clinician or staff will be conducted at the request of the participant (e.g., if developing concerning symptoms or an adverse event) or if needed to clarify study procedures or follow-up symptoms.

10.1.9. *Day 14*

The participant will do the following on Day 14:

- Collect mid-nasal swab for PCR (May be omitted in participants where multiple swabbing is not feasible).
- Complete Daily Survey (online, telephone, or text messaging). This Survey will include confirmation of adherence to study therapy dosing and daily swab collection, concomitant medication review, and symptoms review and objective measures including SpO_2, temperature and pulse.
    -
- *If in DBS Sub-study, collect DBS samples for analysis of concentration of assigned experimental intervention, if not already collected.*

A study team member will be available via Telehealth, text messaging, or telephone to provide support for completion of study procedures.
Clinical outcomes will be confirmed through the electronic health record, if possible.

10.1.10. *Day 28, 56 and 90*

Participants will be contacted on days 28, 56 and 90 to determine well-being. On day 56 a DBS may be collected to determine antibody development

10.1.11. *Participant Reimbursement*

Participants will be reimbursed on Day 14. No reimbursement will be provided to index cases for questionnaire completion and referral of their close contacts. No reimbursement will be provided for unscheduled Telehealth visits requested by the participants for support with study procedures.

11. Study Assessments and Procedures

- Study procedures and their timing are summarized in the SoA.
- Protocol waivers or exemptions are not allowed.
- Immediate safety concerns should be discussed with the sponsor immediately upon occurrence or awareness to determine if the participant should continue or discontinue study treatment.
- Adherence to the study design requirements, including those specified in the SoA, is essential and required for study conduct.
- All baseline evaluations must be completed and reviewed to confirm that potential participants meet all eligibility criteria. The Investigator will maintain a screening log to record details of all participants screened and to confirm eligibility or record reasons for screening failure, as applicable.
- Procedures conducted as part of the participant's routine clinical management and obtained before signing of the informed consent form may be utilized for screening or baseline purposes provided the procedures met the protocol-specified criteria and were performed within the time frame defined in the SoA.
- *Blood samples will only be collected as a part of a sub-study. The maximum amount of blood collected from each participant over the duration of the study, including any extra assessments that may be required, will not exceed 10 mL.*

11.1. *Efficacy Assessments for Brazil*

11.1.1. *Mid-nasal swab or saliva for Brazil*

Health care providers will collect mid-nasal swabs or saliva samples from participants for viral detection at Day 1, Day 3, Day 7, Day 10 and Day 14 (+/-1 day).

Swabs or saliva will be subjected to RNA amplification and tested for SARS-CoV-2 by the local laboratory or by a laboratory designated by it.

11.1.2. *Participant Survey for Brazil*

Participants will be asked to complete Surveys (Daily Survey) that will include questions about symptoms from both the drug regimen, review of concomitant medications.

11.1.2.1. Vital signs assessment

The participant should avoid eating, smoking or exercising for 30 minutes before collecting vital signs measurements. The participant must remain seated and at rest for approximately 5 minutes before the measurement.

11.1.2.2. Heart rate

Heart rate must be measured for a total of 60 seconds, or as stipulated, with pulse oximeter device or Kardiamobile device.

11.1.2.3. Physical examination:

The basic physical exam will be performed at baseline. The following body systems will be examined:

- Cardiovascular examination
- Lungs and chest, including breathing
- Head and neck
- the abdomen
- Musculoskeletal disorders
- Skin
- Neurological assessment

Any new clinically significant physical examination abnormalities identified during the study should be reported as an AE.

11.1.2.4. Cardiac assessment:

The ECG assessment should be performed to check for any changes resulting from COVID-19 and investigational medications. The QTc will be checked on all ECG's.
The participant must rest for a minimum of 5 minutes before the ECG is performed.

11.1.3. *Patient reported outcomes*

Results questionnaires reported by patients (EQ-5D-5L and Dyspnea scale - WURSS-11) will be answered by the participants before the study team carries out any study or clinic evaluation to avoid influencing the participants' responses. Study coordinators will review the participant's responses immediately after the participant completes the questionnaires to ensure that all questions are answered.

Questionnaire EQ-5D-5L: The EQ-5D-5L questionnaire consists of 5 questions, each with five levels (eg, no problems, mild problems, moderate problems, serious problems and extreme problems) representing 5 health domains: mobility, self-care, activities symptoms, pain and / or discomfort, and anxiety and / or depression. The EQ-5D-5L questionnaire also includes a visual analog scale that records the self-assessment of the participant's health status, graded on a scale ranging from 0 to 100, with higher scores indicating greater health-related quality of life. Participants must complete the EQ-5D 5L questionnaire as indicated in the event schedule.

WURSS-11 scale (Wisconsin Upper Respiratory Symptom Survey -11):
https://www.fammed.wisc.edu/files/webfm-uploads/documents/research/wurss-11.pdf

11.2. *Adverse Events for Brazil*

11.2.1. *Definition of Adverse Events for Brazil*

An adverse event is any unfavorable medical occurrence suffered by a patient or a participant in a clinical study who has received a drug that does not necessarily have a causal relationship to that treatment. An AE can therefore be any sign (including an abnormal laboratory finding) or unfavorable and unintended symptom or disease temporally related to the use of a medicated product (under investigation), whether related to the medicated product (under investigation) or not.

That includes:
(1) any new clinical sign or symptom, clinically significant abnormality of physical examination or newly diagnosed event that occurs during the reporting period for AEs, including signs or symptoms associated with an underlying condition that were not present prior to the reporting period. reporting of AEs;
(2) a pre-existing condition that worsened in severity or frequency or changed character after the participant signed the informed consent form during the reporting period for AEs; and
(3) complications that occur as a result of interventions required by the protocol. An AE can arise from any use of the drug under investigation (eg, use outside the indication, use in combination with another drug) and with the use of any route of administration, formulation or dose, including an overdose. AEs can also be any side effects, damage, toxicity or sensitivity reactions that may be experienced by a participant in this clinical study.
For the purposes of this protocol, events that will not be considered AEs include:

- Signs or symptoms expected to fluctuate from a pre-existing medical condition (eg, tremor in a participant with Parkinson's disease; migraine episodes) that did not worsen in severity or frequency or change character during the reporting period for AEs;
- Surgeries or medical procedures are not AEs; however, the clinical condition (new or worse) that led to the surgery or medical procedure is the reported AE (eg, for appendicitis resulting in appendectomy, appendicitis must be reported as the AE);
- Overdose without clinical signs or symptoms;
- Pregnancy.

11.2.2. *Adverse Event Reporting for Brazil*

AEs, including serious adverse events (SAEs), will be collected throughout the study period, from the moment the participant signs the informed consent form and until the End of Study visit. All AEs still present at the time of completion of the study will be monitored by the investigator through contact with the participant until resolution or stabilization, or until the participant loses follow-up and he can no longer be contacted. The result must be documented in the participant's source documents. The investigator must report all AEs that occur after the reporting period specified in the protocol if, according to the investigator's assessment, there is a reasonable possibility that the AEs is related to the product under investigation or any study procedure.

Participants will be asked to complete Surveys (Daily Survey and Exit Contact Survey) that include information on any symptoms that they are experiencing. In addition, AE review by a staff member (via telephone, Telehealth, or text messaging) will be performed.
If the participant reports an AE, it is the investigator's responsibility to obtain sufficient information to assess causality. This may require additional laboratory tests, physical examinations, telephone contacts, etc.

To avoid bias in collecting AEs, participants should be asked to answer a neutral question, such as "How are you feeling?" It is also important to ask the participant in a non-biased way about changes in their health or use of concomitant medication since their last visit. This information must be collected before evaluations are carried out on all study visits. In addition, any symptoms or conditions reported during the assessments and considered clinically significant by the investigator will be assessed as AEs.

11.2.3. *Assessment of Adverse Events for Brazil*

11.2.3.1. Intensity

The medical assessment of intensity will be determined based on the DAIDS grading system: Grade 1 to 4.

A new event will be documented whenever the intensity of an event changes.

It is important to note the distinctions between severe AEs and severe AEs (AEs). Severity is a classification of the intensity of a specific event (such as mild, moderate, severe); however, the event itself may be of relatively secondary clinical significance (such as severe headache). An AE, however, is an AE that meets any of the specified regulatory criteria required for designating severity (eg, a headache may be severe [significantly affects the participant's usual functions], but would not be classified as severe, the unless it met any of the criteria for AEs).

11.2.3.2. Causality and reporting

The investigator will provide a causality assessment for all AEs using their best clinical judgment based on the medical information available about the event being reported. The causality assessment will be reevaluated as new information becomes available. If the investigator's assessment of causality is not reported, the event will be considered "related" until such information is received. Each investigator will assess the degree of relationship between the AE and the drugs under investigation using the following definitions:
Not related: There is no reasonable possibility that the product under investigation caused or contributed to AE.

- The event is related to a different etiology of the drug under investigation, such as underlying disease, study or procedures not included in the study, concomitant drugs or clinical status of the participant
- The timing of the occurrence of AE is not reasonably related to the administration of the study drug
- Related: There is a reasonable possibility that the product under investigation caused or contributed to the AE.
- There is no compatible temporal association between the event and the administration of the drug under investigation
- There is a biologically plausible mechanism by which study treatment may have caused or contributed to AEs
- The event improves or decreases after the study drug is discontinued without the initiation of any specific treatments for the event (withdrawal from exposure) and / or the event recurs or worsens with the reintroduction of study therapy
- The event cannot reasonably be attributed to the concomitant or underlying disease or other medications or procedures

For the purpose of assessing causality, "reasonable possibility" means that, based on the investigator's medical judgment of the available information, there are facts or arguments that suggest a positive causal relationship.

11.2.4. *Serious Adverse Events for Brazil*

An SAE is defined as any untoward medical occurrence that:

- Results in death
- Is life-threatening
- Requires inpatient hospitalization or prolongation of existing hospitalization
- Results in persistent or significant disability/incapacity
- Is an important medical event that may not be immediately life-threatening or result in death or hospitalization but may jeopardize the patient or may require intervention to prevent one of the other outcomes listed in the definition above

All AEs that are recorded will have their severity graded.

11.2.5. *Treatment of Overdose for Brazil*

Overdose of experimental regimens should be managed according to the labeling information (see appendices)
Risks from overdose from placebo pills are expected to be minimal.

11.2.6. *Safety Assessments for Brazil*

Safety will be assessed via participant Surveys, as shown in the SoA.
Participants will be asked to complete Surveys (Daily Survey and Exit Contact Survey) that include questions about their health, healthcare seeking, symptoms, illness within their household, contact, and mobility. Qualifying events will be recorded on the eCRF and reported as AEs, as described in Section 10.3

All AEs that are recorded must have their severity graded. To grade AEs, sites should refer to the DAIDS AE Grading Table, corrected Version 2.1, July 2017, which can be found on the DAIDS Regulatory Support Center website at https://rsc.tech-res.com/docs/default-source/safety/division-of-aids-(daids)-table-for-grading-the-severity-of-adult-and-pediatric-adverse-events-corrected-v-2-1.pdf.

12. Statistical Considerations

12.1. *Sample Size Determination for Brazil*

The sample size for Brazil of 492 patients per arm has been chosen for each experimental group to achieve 90% power with 0.05 two-sided Type 1 error for a pairwise comparison against the control (ascorbic acid) to detect minimum treatment efficacy defined by 27.5 % relative risk reduction (RRR) of preventing hospitalization assuming a control event rate (CER) of 20%.

The table below shows the sample size required at various treatment effects, CER, and drop-out rates for operating characteristics of 90% statistical power and two-sided type I error rate of 5%. Power may be lower depending on CER and drop-out rate (Table 1). As the initial sample size target, 165 per arm has been initially chosen for each experimental group to achieve 90% power with 0.05 two-sided Type 1 error for a pairwise comparison against the control (ascorbic acid) to detect at least 50% treatment efficacy in reducing the progression to LRTI (primary endpoint) assuming a CER of 30% and 5% drop-out rate.

Table 1 Sample Size Per Arm Required to Detect a Relative Risk Reduction of 40% to 60% Under Various Control Event and Drop-out Rates

RRR	Drop-out rate = 0%			Drop-out rate = 5%			Drop-out rate = 20%		
	CER=10%	CER=20%	CER=30%	CER=10%	CER=20%	CER=30%	CER=10%	CER=20%	CER=30%
40%	962	437	261	1013	460	275	1202	546	327
50%	578	263	158	609	277	**165**	723	329	198
60%	375	171	103	395	180	108	469	214	128

Acronyms: CER: Control event rate; RRR: Relative risk reduction.

Sample size calculation was done with the desired operating characteristics of 90% statistical power and 5% type I error rate (two-sided).

As illustrated in the table above, the statistical power will depend heavily on the observed CER. Given uncertainty of the target population, the final target sample size will be re-estimated after four weeks after the first patient is randomized using the observed CER and drop-out rate in the control group as a blinded sample size re-assessment. As it is difficult to predict the enrollment rate, the recruitment rate will be assessed on a weekly basis starting at the end of second week.

The sample size for TOGETHER 3 was determined based on the sample size calculation for TOGETHER 1 (US trial). The initial sample size target for TOGETHER 1 is 165 patients per arm that will achieve 90% power with 0.05 two-sided Type 1 error for a pairwise comparison against the control group (ascorbic acid) to detect treatment effect of 50% relative risk reduction (RRR) in reducing the progression to LRTI (primary endpoint) assuming a control event rate (CER) of 30% and 5% drop-out rate.

12.2. *Populations for Analyses*

For analysis purposes, the following populations are defined and will be described in greater detail in the trial Statistical Analysis Plan:

Population	Description
Intention to Treat (ITT)	All enrolled participants who are randomized into the study: high-risk group for the primary clinical endpoint; high- and low-risk groups (separately) for the primary virologic endpoint.
Safety population	All subjects who received at least 1 dose of study medication
PK evaluable	Participants from the DBS Sub-study with at least 1 interpretable PK sample

DBS: dried blood spot; PK: pharmacokinetic;

12.3. *Statistical Analyses*

The Statistical Analysis Plan will be developed and finalized before database lock and will describe the participant populations to be included in the analyses, the detailed analytical plans

with endpoints and procedures for accounting for missing, unused, and spurious data. An Interim Monitoring Plan will also be developed to describe approaches for re-estimation of sample size and any planned interim analyses. This section presents a brief summary of the planned statistical analyses of the primary and secondary endpoints (these are listed in Section 0).

12.3.1. *Efficacy Analyses*

Demographic characteristics (age, sex, race) of each study group will be tabulated.

The mean age (plus range and standard deviation) by sex of the enrolled participants, as a whole and per group, will be calculated.

Primary analyses:
The primary analyses will be conducted on the ITT population overall and by cohort. Participants randomized to each active arm will be compared to participants randomized to placebo (ascorbic acid). The primary analysis will make use of all participants randomized to placebo, whether contemporaneously enrolled or not using an Empirical Bayesian information borrowing method (described below). Due to anticipated heterogeneity in risk of disease progression, pre-specified baseline variables, including age, weight, and days of symptoms at time of enrollment will be included in the model to increase precision.

Sensitivity analysis:
The primary analyses will be repeated replacing randomization arm by actual treatment to account for possible off-label use of and noncompliance to the investigational products.

Subgroup analyses:
All subgroup analyses will be pre-specified in the Statistical Analysis Plan. Any further subgroups will be considered ad hoc.

Empirical Bayesian information borrowing method:
In the case that a new experimental treatment is added into this platform trial, there will already be data collected from patients who are enrolled prior (past data). To incorporate the past control data with the concurrent control data from patients who are concurrently enrolled, empirical Bayesian information borrowing method will be used[32]. Instead of using concurrent data only, combining past data with the concurrent data can potentially be advantageous in terms of statistical power and lower number of participants that are needed to be randomized to the control arm. This is particularly important for possible improved ethics and feasibility for the context of conducting a platform trial for SARS-CoV-2. Additionally, in the case that relevant external trial data become available, this empirical information borrowing method may be used. The DSMB, which will contain clinical experts, will make on whether to include or ignore the external trial data. The Study Statistician will assess the similarities of the external trial(s) to this clinical trial in terms of eligibility criteria, trial location, data collection procedures, and the reliability and comprehensiveness of the available dataset. If deemed appropriate, the empirical Bayesian information borrowing method will be used to determine the degree of the information that can be “borrowed” from the external dataset and be used for the statistical comparison.

As there are multiple randomized clinical trials that are either ongoing or being planned right now for SARS-CoV-2 with potentially relevant interventions, it is important to plan for an approach, such as this empirical Bayesian borrowing method, that can potentially incorporate these external data with the internal data. An important feature of this method is the avoidance of use of any subjective or informative prior distributions that may become a point of dispute in certain schools of thought.

The full technical details on the empirical Bayesian information borrowing method will be described in the Statistical Analysis Plan.

Missing Data:
Due to the design of the study and retention activities, we expect to be able measure outcomes on all participants. However, in the unlikely event of a missing test result, the missing data will be imputed.

Interim Analysis:
The DSMB will decide on a possible interim analysis during the trial after the sample size re-assessment based on the information on the observed recruitment rate, the CER, the drop-out rate, and the final sample size target, if applicable. The decision for interim analysis will be made in a blinded manner (e.g., based on pooled number of events). The interim monitoring plan (written by the Study Statistician) will define monitoring bounds to maintain the two-sided type I error rate at the desired 5% (e.g. 97.5% or higher probability of superiority over the control group). Should new experimental candidates be added during the trial, allocation ratios will be adapted to favor the new arms.

12.3.2. *Secondary endpoints*
All secondary endpoints will be assessed in the Intention-to-Treat population overall and by cohort.

12.3.2.1. Safety Analyses
All safety analyses will be performed on the Intention-to-Treat population. AEs will be compared by study group.

12.3.2.2. Hospitalization
Hospitalization rates between the groups will be compared using logistic regression stratified by site. Number of days hospitalized will be described graphically and by median and interquartile rage.

12.3.2.3. Symptom Resolution
Days with fever after randomization, respiratory symptoms (ordinal outcome) after randomization after randomization will be modeled using proportional odds regression method stratified by site.

The procedure assumes multinomial distributions for the marginal (i.e., time-specific) univariate ordinal responses and uses a working correlation structure to account for within-subject association between the repeated measures. Time-dependent covariates (e.g. ordinal responses at previous timepoints) will be included the statistical model.

12.3.3. *Pharmacokinetic Analysis*
Sparse PK from DBS will be analyzed using standard population PK analysis methodologies using standard software such as NONMEM® V7.4 or Phoenix NLME V8.2.

12.3.4. *Exploratory Exposure-Response Analyses*
PK-evaluable participants will have post-hoc individual concentration profiles and exposure estimates determined for exploratory exposure-response analyses against primary and secondary efficacy and safety endpoints. Exploratory PK/pharmacodynamic analyses will be performed as the data allow.

12.3.5. *Combined Study Analysis*

This protocol is being published as a model protocol for other institutions to consider as they undertake studying treatments to prevent development of LRTI among outpatients with SARS-CoV-2 infection. It is hoped that individual patient data from similar studies can be pooled into a combined study analysis. De-identified data from the present study will be made available for these purposes in accordance with the funder's open access policy (https://www.gatesfoundation.org/how-we-work/general-information/open-access-policy).

13. Reimbursement

13.1. *Reimbursement in Brazil*

No reimbursement will be made in Brazil.

14. References

1. Huang C, Wang Y, Li X, et al. Clinical features of patients infected with 2019 novel coronavirus in Wuhan, China. *Lancet* 2020; **395**(10223): 497-506.
2. Zhu N, Zhang D, Wang W, et al. A Novel Coronavirus from Patients with Pneumonia in China, 2019. *N Engl J Med* 2020; **382**(8): 727-33.
3. Chen N, Zhou M, Dong X, et al. Epidemiological and clinical characteristics of 99 cases of 2019 novel coronavirus pneumonia in Wuhan, China: a descriptive study. *Lancet* 2020; **395**(10223): 507-13.
4. Release WN. Statement on the second meeting of the International Health Regulations (2005) Emergency Committee regarding the outbreak of novel coronavirus (2019-nCoV). 30 January 2020 2020. https://www.who.int/news-room/detail/30-01-2020-statement-on-the-second-meeting-of-the-international-health-regulations-(2005)-emergency-committee-regarding-the-outbreak-of-novel-coronavirus-(2019-ncov).
5. Cai J, Xu J, Lin D, et al. A Case Series of children with 2019 novel coronavirus infection: clinical and epidemiological features. *Clin Infect Dis* 2020.
6. Linton NM, Kobayashi T, Yang Y, et al. Incubation Period and Other Epidemiological Characteristics of 2019 Novel Coronavirus Infections with Right Truncation: A Statistical Analysis of Publicly Available Case Data. *J Clin Med* 2020; **9**(2).
7. Release WN. Shortage of personal protective equipment endangering health workers worldwide. 2020. https://www.who.int/news-room/detail/03-03-2020-shortage-of-personal-protective-equipment-endangering-health-workers-worldwide.
8. Chen J. Pathogenicity and transmissibility of 2019-nCoV-A quick overview and comparison with other emerging viruses. *Microbes Infect* 2020; **22**(2): 69-71.
9. Hoffmann M, Kleine-Weber H, Schroeder S, et al. SARS-CoV-2 Cell Entry Depends on ACE2 and TMPRSS2 and Is Blocked by a Clinically Proven Protease Inhibitor. *Cell* 2020.
10. Li CC, Wang XJ, Wang HR. Repurposing host-based therapeutics to control coronavirus and influenza virus. *Drug Discov Today* 2019; **24**(3): 726-36.
11. Slater AF. Chloroquine: mechanism of drug action and resistance in Plasmodium falciparum. *Pharmacol Ther* 1993; **57**(2-3): 203-35.
12. Mackenzie AH. An appraisal of chloroquine. *Arthritis Rheum* 1970; **13**(3): 280-91.
13. Shippey EA, Wagler VD, Collamer AN. Hydroxychloroquine: An old drug with new relevance. *Cleve Clin J Med* 2018; **85**(6): 459-67.
14. Schrezenmeier E, Dorner T. Mechanisms of action of hydroxychloroquine and chloroquine: implications for rheumatology. *Nat Rev Rheumatol* 2020; **16**(3): 155-66.
15. Rolain JM, Colson P, Raoult D. Recycling of chloroquine and its hydroxyl analogue to face bacterial, fungal and viral infections in the 21st century. *Int J Antimicrob Agents* 2007; **30**(4): 297-308.
16. Savarino A, Shytaj IL. Chloroquine and beyond: exploring anti-rheumatic drugs to reduce immune hyperactivation in HIV/AIDS. *Retrovirology* 2015; **12**: 51.
17. Keyaerts E, Vijgen L, Maes P, Neyts J, Van Ranst M. In vitro inhibition of severe acute respiratory syndrome coronavirus by chloroquine. *Biochem Biophys Res Commun* 2004; **323**(1): 264-8.
18. Vincent MJ, Bergeron E, Benjannet S, et al. Chloroquine is a potent inhibitor of SARS coronavirus infection and spread. *Virol J* 2005; **2**: 69.
19. Wang M, Cao R, Zhang L, et al. Remdesivir and chloroquine effectively inhibit the recently emerged novel coronavirus (2019-nCoV) in vitro. *Cell Res* 2020; **30**(3): 269-71.
20. Yao X, Ye F, Zhang M, et al. In Vitro Antiviral Activity and Projection of Optimized Dosing Design of Hydroxychloroquine for the Treatment of Severe Acute Respiratory Syndrome Coronavirus 2 (SARS-CoV-2). *Clin Infect Dis* 2020.

21. Gao J, Tian Z, Yang X. Breakthrough: Chloroquine phosphate has shown apparent efficacy in treatment of COVID-19 associated pneumonia in clinical studies. *Biosci Trends* 2020; **14**(1): 72-3.
22. Gautret P, Lagier JC, Parola P, et al. Hydroxychloroquine and azithromycin as a treatment of COVID-19: results of an open-label non-randomized clinical trial. *Int J Antimicrob Agents* 2020: 105949.
23. Menzel M, Akbarshahi H, Bjermer L, Uller L. Azithromycin induces anti-viral effects in cultured bronchial epithelial cells from COPD patients. *Sci Rep* 2016; **6**: 28698.
24. Poschet JF, Perkett EA, Timmins GS, et al. Azithromycin and ciprofloxacin have a chloroquine-like effect on respiratory epithelial cells. 2020. bioRxiv preprint. doi: https://doi.org/10.1101/2020.03.29.008631.
25. Belouzard S, Millet JK, Licitra BN, Whittaker GR. Mechanisms of coronavirus cell entry mediated by the viral spike protein. *Viruses* 2012; **4**(6): 1011-33.
26. Wrapp D, Wang N, Corbett KS, et al. Cryo-EM structure of the 2019-nCoV spike in the prefusion conformation. *Science* 2020; **367**(6483): 1260-3.
27. Furst DE, Lindsley H, Baethge B, et al. Dose-loading with hydroxychloroquine improves the rate of response in early, active rheumatoid arthritis: a randomized, double-blind six-week trial with eighteen-week extension. *Arthritis Rheum* 1999; **42**(2): 357-65.
28. Collins KP, Jackson KM, Gustafson DL. Hydroxychloroquine: A Physiologically-Based Pharmacokinetic Model in the Context of Cancer-Related Autophagy Modulation. *J Pharmacol Exp Ther* 2018; **365**(3): 447-59.
29. Tett SE, Cutler DJ, Day RO, Brown KF. Bioavailability of hydroxychloroquine tablets in healthy volunteers. *Br J Clin Pharmacol* 1989; **27**(6): 771-9.
30. Graat JM, Schouten EG, Kok FJ. Effect of daily vitamin E and multivitamin-mineral supplementation on acute respiratory tract infections in elderly persons: a randomized controlled trial. *JAMA* 2002; **288**(6): 715-21.
31. Packaging UM. Biological Substance Category B. 2020. https://www.un3373.com/category-biological-substances/category-b.
32. Schoenfeld D, Zheng H, Finkelstein D. Bayesian design using adult data to augment pediatric trials. *Clinical Trials* 2009; **6**: 297-304.
