# Supplementary material for: Effect of Early Treatment With Hydroxychloroquine or Lopinavir and Ritonavir on Risk of Hospitalization Among Patients With COVID-19: The TOGETHER Randomized Clinical Trial
Source: JAMA Netw Open. 2021 Apr 22;4(4):e216468. doi: 10.1001/jamanetworkopen.2021.6468 (PMC8063069; doi:10.1001/jamanetworkopen.2021.6468)
Supplement: Supplement 2. — eAppendix. Trial Organization eTable 1. Summary of Cox Proportional Hazard Regression Model for COVID-19 And All-Cause Hospitalization eTable 2. Summary of Sub-Group Analyses for COVID-19 And All-Cause Hospitalization Outcomes eTable 3. Summary of Mixed-Effect Logistic Regression Model for Viral Suppression eTable 4. Summary of Sub-Group Analyses on Viral Suppression, Intention to Treat Analysis eTable 5. Summary of Cox Proportional Hazard Regression Model for Time to COVID-19 Related Symptoms Resolution [file jamanetwopen-e216468-s002.pdf]

Supplemental Online Content

Reis G, Moreira Silva EAS, Medeiros Silva DC, et al; TOGETHER Investigators. Effect of early treatment with hydroxychloroquine or lopinavir and ritonavir on risk of hospitalization among patients with COVID-19: the TOGETHER randomized clinical trial. *JAMA Netw Open*. 2021;4(4):e216468. doi:10.1001/jamanetworkopen.2021.6468

eAppendix. Trial Organization

eTable 1. Summary of Cox Proportional Hazard Regression Model for COVID-19 And All-Cause Hospitalization

eTable 2. Summary of Sub-Group Analyses for COVID-19 And All-Cause Hospitalization Outcomes

eTable 3. Summary of Mixed-Effect Logistic Regression Model for Viral Suppression

eTable 4. Summary of Sub-Group Analyses on Viral Suppression, Intention to Treat Analysis

eTable 5. Summary of Cox Proportional Hazard Regression Model for Time to COVID-19 Related Symptoms Resolution

This supplemental material has been provided by the authors to give readers additional information about their work.

© 2021 Reis G et al. *JAMA Network Open*.

eAppendix. Trial Organization

The COVID-19 TOGETHER initiative was designed to evaluate repurposed treatments for COVID-19 disease through an adaptive trial design in three arms being conducted at United States, Brazil and South Africa. The Brazilian TOGETHER arm was purported by a network of primary care research centers from the state of Minas Gerais, Brazil, devoted to a comprehensive evaluation and treatment of patient with COVID-19. The trial was fully integrated with local public health authorities (Brazilian Unified Health System – SUS) as part of coping strategy for COVID-19 pandemic. Namely, the main institutions involved were: Cardresearch – Cardiologia Assistencial e de Pesquisa leading Brazilian Arm, University of Washington leading United Stated arm and Stellenbosh University leading South Africa Arm. This initiative was funded by Bill & Melinda Gates Foundation through University of Washington subaward number UWSC11903. The Executive Committee had one member from each institution, and Steering Committee was comprised also of members from all institutions.

Brazilian Executive Committee

Gilmar Reis[1,2], Eduardo AAM Silva[1,2], Daniela CM Silva[1,2], Castilho VQ Santos[2], Ana PFG Almeida[3], Adhemar DF Neto[4], Leonardo CM Savassi[5], Maria IC Simplicio[1], Edward J Mills[6,7]

Steering and Executive Committee

Jared M Baeten[8], Gilmar Reis[1,2], Lehana Thabane[6], Jay JH Park[7,9], Edward J Mills[6,7], Eric H Decloedt[10], Christine M Johnston[8]

[1]Cardresearch – Cardiologia Assistencial e de Pesquisa, Belo Horizonte, Brazil; [2]Pontificia Universidade Catolica de Minas Gerais, Belo Horizonte; [3]Montes Claros State University, Montes Claros, Brazil; [4]Federal University of Juiz de Fora, Governador Valadares, Brazil; [5]Federal University of Ouro Preto, Ouro Preto, Brazil; [6]McMaster University, Hamilton, Canada, [7]Cytel Health, Vancouver, Canada, [8]University of Washington, Seattle, USA; [9]University of British Columbia, Vancouver, Canada; [10]Stellenbosch University, Cape Town, South Africa;

Data Safety and Monitoring Board

David V. Glidden[1] (chair), Robert Coombs[2], Michael Boeckh[3], Israel Molina Romero[4], Gary Maartens[5]

[1]Department of Biostatistics and Epidemiology, School of Medicine, University of California, San Francisco, USA; [2]Department of Laboratory Medicine, Virology Division, Clinical Retrovirus Laboratory, University of Washington, Seattle, USA; [3]Vaccine and Infectious Disease Division, Clinical Research Division, Fred Hutch, Seattle, USA; [4]Infectious Disease Department, Vall d' Hebron University Hospital, Universitat Autònoma de Barcelona, Barcelona, Spain and Imunopathology Laboratory, Feceral University of Ouro Preto, Ouro Preto, Brazil; [5]Clinical Pathology Laboratory, University of Cape Town, Cape town, South Africa.

Clinical Events Classification Committee

Gilmar Reis[1,2] (chair), Luciene B Ribeiro[1] (adjudicator), Thiago Santiago Ferreira[1] (adjudicator), Vinicius Alves Correa (adjudicator), Mayra Stefane Silva dos Santos[1] (operations manager)

[1]Cardresearch – Cardiologia Assistencial e de Pesquisa, Belo Horizonte, Brazil; [2]Pontificia Universidade Catolica de Minas Gerais, Belo Horizonte, Brazil;

Trial Operations

Maria Izabel Santos Simplicio[1] (site manager), Mayra Stefane Silva dos Santos (site monitoring)[1], Thais Campos Siqueira[1] (site monitoring), Carla Stefany Oliveira França[1] (data management), Aline Cruz Milagres[1] (data management), Luciene Barra Ribeiro[1] (regulatory affairs manager), Layla Fortes Pereira[1] (regulatory affairs), Camila Davi Rodrigues Kattah[1] (pharmaceutical operations manager), Davi Rodrigues Kattah[1] (pharmaceutical operations), Lineria Honorio de Morais Suterio[1] (pharmaceutical operations).

[1]Cardresearch – Cardiologia Assistencial e de Pesquisa, Belo Horizonte, Brazil; [2]McMaster University, Hamilton, Canada, [3]Cytel Health, Vancouver, Canada

Participating Enrolling Centres:

Included below are representatives from the enrolling centers at participating cities that enrolled at least 1 patient. Centres are listed in order of enrollment contribution. All study sites were located in the State of Minas Gerais, Brazil.

Cardresearch – Cardiologia Assistencial e de Pesquisa

Ana Flavia Gomes Viana, Carla Stefany Oliveira Franca, Gabriela Alves de Oliveira, Gilmar Reis, Guilherme Henrique Ferreira Pinto, Laura Campos Oliveira, Layla Fortes Pereira, Lorena Mesquita Alves, Lorena Mesquita Alves, Luana Rosa Rodrigues, Luciene Barra Ribeiro, Luiza Ramos Soares de Oliveira, Maria Izabel Campos Simplicio, Maria Paula Andrade Rodrigues Machado , Matheus Veloso Magalhães, Mayra Estefane Silva dos Santos, Mayron Douglas Viegas Freitas, Rosemary Oliveira, Sílvia Goulart Curi, Thais Campos Siqueira, Thiago Santiago Ferreira, Vânia Regina Campos

City of Sete Lagoas

Ana Carolina Matos Ferreira, Ana Caroline Santos Silva, Ana Clara Ramos Ferreira, Arthur Henrique Abreu Rocha, Arthur Mendes Gasperini, Castilho Vitor Quirino dos Santos, Daniel Domingues Barbosa, Eduardo Augusto dos Santos Medeiros Silva, Emanuela Pontes Pereira Silveira, Emanuelle Cristina Soares Gonçalves, Gabriel Corrêa Costa, Gabriela Martins Costa, Geraldo César Barroso de Souza, Guilherme Aurélio de Oliveira Alves, Jéssica Aparecida Da Silva Ribeiro, Kenia Sthephane Guimaraes Goncalves, Karine Fernanda Lopes dos Santos, Lara Silveira Marques, Marcella Alves Rodrigues, Marcelo Rodrigues da Costa Fernandes, Maria Eduarda Guimarães de Sousa, Maria Thereza Figueiredo Belém Calazans, Pedro Henrique Arcanjo Alvarenga, Vinicius Alves Correa,

© 2021 Reis G et al. *JAMA Network Open.*

Vinícius Gustavo de Carvalho Moura, Vitória Aparecida Cunha, Vitória Helena de Souza Campos, Viviane Costa Santos, Quimberli Vassinave Cujuí

City of Montes Claros

Alexandre Oliveira Sidônio, Ana Maria Ribeiro Nogueira, Ana Paula Figueiredo Guimarães de Almeida, Anne Ribeiro Magalhães, Artur Pimenta Ribeiro, Caio Gonçalves Nogueira, Caroline de Sá Rocha Ferreira, Clarice Ribeiro de Oliveira Matos, Cleide Rocha Veloso, Eloange Alkmim Lima Muniz, Fernanda Dantas Carvalho, Igor Antônio Tolentino Narciso, Jeferson Augusto Brito Souza Barbosa, João Pedro Silveira Gonçalves, Jorge Fernando Rocha Veloso, Karolina Campos Sampaio Lopes Campos, Lorena Soares David, Luciana Santana Ribeiro, Ludimila Pereira de Souza, Maria Claudia Prates da Costa, Maria de Fátima César Lima, Maria Fernanda Batista Pereira de Matos, Maria Izabel Silveira Gonçalves, Maria Luiza Vianna Meira, Mayra Darlliane Loiola Silva, Samuel de Paiva Oliveira, Thiago Rodrigues Ferro, Walfredo Gonçalves de Quadros Júnior

City of Governador Valadares

Adhemar Dias de Figueiredo Neto, Aline do Carmo Rosa, Ana Carolina Lima Delgado, Ana Cláudia Barbosa Marinho, Ana Paula Vilas Boas Wheberth, Arthur Henrique Nunes Leite Oliveira, Caio de Cássio Bernardes, Camila Borba Pereira, Daminiana Rogai Siqueira, Daniel Viana Gonçalves Glória, Emanuel Peixoto Pinto, Felipe Coelho Soares de Oliveira, Felipe Fraga Damaceno, Guilherme Rhis, Igor Brandão Rocha, Igor Pereira Matos de Oliveira, José Marques Pio II, Lara Oliveira Lopes, Larissa Cruz Terra, Laura Lima Vargas, Lilia Dayana de Castro, Luísa Di Mambro Rezende, Luiz Eduardo Coelho Fava, Maria tereza Cardozo Victal, Mariana Waquimaker Figueiredo, Marina Lacerda Marques, Mateus Pereira santos Souza, Mayara de Freitas Reis, Monique Gonçalves Flor, Neíla Rodrigues Vargas de Paula, Pamela de Sousa dias Demuner, Priscilla Alves Meira, Rafaella Rosa de Oliveira Fernandes, Ramail Santos Pouzas, Roberta Coelho De Marco, Rodrigo Brandão Alves, Ronan Figueiredo, Sabrina Stefany da Silva Souza, Sarah Silva Ferraz, Tainara Cristina Silva Idelfonso, Tânia Santos Portugal, Thiago Antônio da Silva Fontoura, Tiago almeida Catombé, Vitor Guimarães Lage

City of Ibirité

Alexandre Moreira Sales, Aline Cruz Milagres, André Henrique de Sousa Oliveira, Cristiano Afonso de Souza Pereira, Daniela Pinheiro de Matos, Élida Juliane Rezende Moreira, Fabiana Marçal Ferreira, Fernanda Mecchi, Gabriel Milagres de Almeida, Geisiane Almeida da Cruz, Geisyane Rosa Gomes, Guilherme Dias Ferreira, Hugo Pimenta Ferreira, João Paulo Braga Fernandes Sobreira, Laylla Michelle Pereira, Leonardo Cançado Monteiro Savassi, Paloma dos Reis Freitas de Morais, Paula Junqueira

City of Betim

Alberto Andrade Horta Dumont, Daniela Carla Medeiros, Hilton Soares de Oliveira, Isnard Fernandes de Souza Neto, João Pedro Machado Cardoso Nascimento, Laylla Michelle Pereira, Leandro Hugo Teles da Silva, Livian Pereira Ribeiro, Patrícia Sant'Ana de Assis Silva, Pedro Henrique Vimieiro da Silva, Thariny Marie de Franca Xavier

City of Nova Lima

Aline Lucia de Jesus Martins, Daniel Vitor Loiola de Aguiar, Maria Paula Andrade Rodrigues Machado, Tainara Silva Vieira, Thiago Santiago Ferreira,

City of Itaúna

Andressa Corradi Sousa Teixeira, Camila Mendes Freitas Moreira, Isnard fernandes de Souza Neto, Leandro Hugo Teles da Silva

Public health authorities and mayors
We are in debt with the following local public health authorities and mayors (listed by enrollment):

City of Sete Lagoas
Duílio de Castro Faria (Mayor), Flávio Pimenta Silveira (public health authority), Sueli Barbosa dos Santos Lacerda (public health epidemiology)

City of Betim
Vittorio Medioli (Mayor), Guilherme Carvalho da Paixão (public health authority),

City of Montes Claros
Humberto Guimarães Souto (Mayor), Dulce Pimenta Gonçalves (public health authority)

City of Governador Valadares
André Luiz Coelho Merlo (Mayor), Edna Gomes Leite (public health authority)

City of Ibirité
William Parreira Duarte (Mayor), Carina Bitarães (public health authority)

City of Nova Lima
Vitor Penido de Barros (Mayor), José Roberto Lintz Machado (public health authority)

City of Itaúna
Neider Moreira de Faria (Mayor), Fernando Meira de Faria (public health authority)

© 2021 Reis G et al. *JAMA Network Open.*

Sponsorship and Data Sharing Statement

Sponsorship

The trial was designed by the authors and sponsored by the Bill & Mellinda Gates Foundation through University of Washington subaward number UWSC11903. Trial was overseen by the authors, university of Washington and Cytel Health Canada. An independent data safety monitoring board analysed the data as per DMSB monitoring plan, which included futility analysis. The sponsor did not have any influence on trial conduct, data collection, analysis and report, including publication. All participating research centers were included in the study. The Executive Committee wrote the first draft of the manuscript and tables and figures. The manuscript was subsequently revised and approved by all the authors and research network members, who agreed to submit the manuscript for publication.

Data Sharing Statement

The study protocol and clinical study plan are will be in the web-appendix. Anonymized participant data will be made available when the trial is complete, upon requests directed to the corresponding author. Proposals will be reviewed and approved by the sponsor, investigator, and collaborators on the basis of scientific merit. After approval of a proposal, data can be shared through a secure online platform after signing a data access agreement. All data will be made available for a minimum of 5 years from the end of the trial.

© 2021 Reis G et al. *JAMA Network Open.*

Supplementary Materials for Statistical Analyses

Hospitalization analyses

eTable 1: Summary of Cox Proportional Hazard Regression Model for COVID-19 and All-Cause Hospitalization

Outcome	Treatment vs placebo	HR (95% CI)	exp(coef)	se(coef)	P-value
Intention to treat analysis					
Covid-related	HCQ	0.76 [0.30; 1.88]	0.75739718	0.46466457	0.54984269
Covid-related	LPV/r	1.17 [0.53; 2.59]	1.17354209	0.4029142	0.69123996
All cause	HCQ	0.96 [0.42; 2.17]	0.95550397	0.4174276	0.91317067
All cause	LPV/r	1.23 [0.58; 2.60]	1.22832319	0.38188398	0.59022205
Per-protocol analysis					
Covid-related	HCQ	0.91 [0.33; 2.52]	0.91364843	0.51755139	0.86147752
Covid-related	LPV/r	1.84 [0.77; 4.39]	1.84246638	0.44321386	0.16795516
All cause	HCQ	1.31 [0.52; 3.31]	1.30647261	0.47434445	0.57304029
All cause	LPV/r	2.10 [0.90; 4.91]	2.10320735	0.43302335	0.08599504

© 2021 Reis G et al. *JAMA Network Open.*

eTable 2: Summary of Sub-Group Analyses for COVID-19 and All-Cause Hospitalization Outcomes

Analysis	Outcome	Treatment	Factor	Subgroup	N_Placebo	N_Trt	N_events_Placebo	N_events_Trt	HR (95% CI)
ITT	Covid-related	HCQ	Age	<50	72	77	3	0	0.00 [0.00; Inf]
ITT	Covid-related	HCQ	Age	>=50	150	133	8	8	1.11 [0.42; 2.97]
ITT	Covid-related	HCQ	Sex	F	121	122	4	5	1.22 [0.33; 4.56]
ITT	Covid-related	HCQ	Sex	M	106	92	7	3	0.48 [0.12; 1.87]
ITT	Covid-related	HCQ	Diabetes	N	180	173	9	5	0.56 [0.19; 1.68]
ITT	Covid-related	HCQ	Diabetes	Y	47	40	2	3	1.78 [0.30; 10.66]
ITT	Covid-related	HCQ	Cardiac Disease	N	118	111	4	4	1.06 [0.26; 4.22]
ITT	Covid-related	HCQ	Cardiac Disease	Y	109	103	7	4	0.59 [0.17; 2.01]
ITT	Covid-related	HCQ	Lung Disease	N	207	190	10	8	0.86 [0.34; 2.17]
ITT	Covid-related	HCQ	Lung Disease	Y	20	24	1	0	0.00 [0.00; Inf]
ITT	Covid-related	HCQ	Symptom Onset	< 120 hours	40	37	0	1	644107020.33 [0.00; Inf]
ITT	Covid-related	HCQ	Symptom Onset	>= 120 hours	187	177	11	7	0.65 [0.25; 1.68]
ITT	Covid-related	LPV/r	Age	<50	72	89	3	3	0.78 [0.16; 3.87]
ITT	Covid-related	LPV/r	Age	>=50	150	149	8	11	1.39 [0.56; 3.46]
ITT	Covid-related	LPV/r	Sex	F	121	134	4	3	0.66 [0.15; 2.97]
ITT	Covid-related	LPV/r	Sex	M	106	110	7	11	1.53 [0.59; 3.94]
ITT	Covid-related	LPV/r	Diabetes	N	180	200	9	9	0.89 [0.35; 2.24]
ITT	Covid-related	LPV/r	Diabetes	Y	47	43	2	5	2.77 [0.54; 14.28]
ITT	Covid-related	LPV/r	Cardiac Disease	N	118	111	4	6	1.59 [0.45; 5.62]
ITT	Covid-related	LPV/r	Cardiac Disease	Y	109	133	7	8	0.92 [0.33; 2.55]
ITT	Covid-related	LPV/r	Lung Disease	N	207	229	10	13	1.16 [0.51; 2.64]
ITT	Covid-related	LPV/r	Lung Disease	Y	20	15	1	1	1.39 [0.09; 22.27]
ITT	Covid-related	LPV/r	Symptom Onset	< 120 hours	40	34	0	2	719455295.05 [0.00; Inf]
ITT	Covid-related	LPV/r	Symptom Onset	>= 120 hours	187	210	11	12	0.96 [0.42; 2.17]

© 2021 Reis G et al. *JAMA Network Open.*

Analysis	Outcome	Treatment	Factor	Subgroup	N_Placebo	N_Trt	N_events_Placebo	N_events_Trt	HR (95% CI)
ITT	All cause	HCQ	Age	<50	72	77	3	2	0.60 [0.10; 3.60]
ITT	All cause	HCQ	Age	>=50	150	133	9	9	1.12 [0.44; 2.81]
ITT	All cause	HCQ	Sex	F	121	122	5	7	1.37 [0.44; 4.33]
ITT	All cause	HCQ	Sex	M	106	92	7	4	0.64 [0.19; 2.20]
ITT	All cause	HCQ	Diabetes	N	180	173	10	7	0.71 [0.27; 1.87]
ITT	All cause	HCQ	Diabetes	Y	47	40	2	4	2.37 [0.43; 12.95]
ITT	All cause	HCQ	Cardiac Disease	N	118	111	4	5	1.32 [0.35; 4.91]
ITT	All cause	HCQ	Cardiac Disease	Y	109	103	8	6	0.77 [0.27; 2.22]
ITT	All cause	HCQ	Lung Disease	N	207	190	11	11	1.07 [0.46; 2.47]
ITT	All cause	HCQ	Lung Disease	Y	20	24	1	0	0.00 [0.00; Inf]
ITT	All cause	HCQ	Symptom Onset	< 120 hours	40	37	0	2	653712362.41 [0.00; Inf]
ITT	All cause	HCQ	Symptom Onset	>= 120 hours	187	177	12	9	0.77 [0.32; 1.83]
ITT	All cause	LPV/r	Age	<50	72	89	3	3	0.78 [0.16; 3.87]
ITT	All cause	LPV/r	Age	>=50	150	149	9	13	1.46 [0.62; 3.42]
ITT	All cause	LPV/r	Sex	F	121	134	5	4	0.71 [0.19; 2.64]
ITT	All cause	LPV/r	Sex	M	106	110	7	12	1.66 [0.65; 4.22]
ITT	All cause	LPV/r	Diabetes	N	180	200	10	11	0.98 [0.41; 2.30]
ITT	All cause	LPV/r	Diabetes	Y	47	43	2	5	2.77 [0.54; 14.28]
ITT	All cause	LPV/r	Cardiac Disease	N	118	111	4	6	1.59 [0.45; 5.62]
ITT	All cause	LPV/r	Cardiac Disease	Y	109	133	8	10	1.01 [0.40; 2.55]
ITT	All cause	LPV/r	Lung Disease	N	207	229	11	14	1.13 [0.52; 2.50]
ITT	All cause	LPV/r	Lung Disease	Y	20	15	1	2	2.75 [0.25; 30.28]
ITT	All cause	LPV/r	Symptom Onset	< 120 hours	40	34	0	2	719455295.05 [0.00; Inf]
ITT	All cause	LPV/r	Symptom Onset	>= 120 hours	187	210	12	14	1.02 [0.47; 2.21]
PP	Covid-related	HCQ	Age	<50	66	72	2	0	0.00 [0.00; Inf]
PP	Covid-related	HCQ	Age	>=50	137	122	6	7	1.31 [0.44; 3.88]

© 2021 Reis G et al. *JAMA Network Open.*

Analysis	Outcome	Treatment	Factor	Subgroup	N_Placebo	N_Trt	N_events_Placebo	N_events_Trt	HR (95% CI)
PP	Covid-related	HCQ	Sex	F	110	110	4	4	0.99 [0.25; 3.98]
PP	Covid-related	HCQ	Sex	M	98	88	4	3	0.83 [0.19; 3.71]
PP	Covid-related	HCQ	Diabetes	N	163	159	7	4	0.58 [0.17; 1.97]
PP	Covid-related	HCQ	Diabetes	Y	45	38	1	3	3.68 [0.38; 35.43]
PP	Covid-related	HCQ	Cardiac Disease	N	106	102	2	3	1.57 [0.26; 9.39]
PP	Covid-related	HCQ	Cardiac Disease	Y	102	96	6	4	0.70 [0.20; 2.46]
PP	Covid-related	HCQ	Lung Disease	N	189	176	7	7	1.07 [0.38; 3.05]
PP	Covid-related	HCQ	Lung Disease	Y	19	22	1	0	0.00 [0.00; Inf]
PP	Covid-related	HCQ	Symptom Onset	< 120 hours	36	34	0	1	630105398.39 [0.00; Inf]
PP	Covid-related	HCQ	Symptom Onset	>= 120 hours	172	164	8	6	0.77 [0.27; 2.23]
PP	Covid-related	LPV/r	Age	<50	66	75	2	3	1.31 [0.22; 7.85]
PP	Covid-related	LPV/r	Age	>=50	137	119	6	11	2.17 [0.80; 5.86]
PP	Covid-related	LPV/r	Sex	F	110	110	4	3	0.75 [0.17; 3.34]
PP	Covid-related	LPV/r	Sex	M	98	90	4	11	3.11 [0.99; 9.77]
PP	Covid-related	LPV/r	Diabetes	N	163	161	7	9	1.31 [0.49; 3.51]
PP	Covid-related	LPV/r	Diabetes	Y	45	38	1	5	6.19 [0.72; 52.96]
PP	Covid-related	LPV/r	Cardiac Disease	N	106	91	2	6	3.56 [0.72; 17.65]
PP	Covid-related	LPV/r	Cardiac Disease	Y	102	109	6	8	1.26 [0.44; 3.62]
PP	Covid-related	LPV/r	Lung Disease	N	189	187	7	13	1.89 [0.76; 4.75]
PP	Covid-related	LPV/r	Lung Disease	Y	19	13	1	1	1.54 [0.10; 24.65]
PP	Covid-related	LPV/r	Symptom Onset	< 120 hours	36	30	0	2	738063520.52 [0.00; Inf]
PP	Covid-related	LPV/r	Symptom Onset	>= 120 hours	172	170	8	12	1.53 [0.62; 3.74]
PP	All cause	HCQ	Age	<50	66	72	2	2	0.90 [0.13; 6.41]
PP	All cause	HCQ	Age	>=50	137	122	6	8	1.49 [0.52; 4.30]
PP	All cause	HCQ	Sex	F	110	110	4	6	1.49 [0.42; 5.30]
PP	All cause	HCQ	Sex	M	98	88	4	4	1.11 [0.28; 4.43]

© 2021 Reis G et al. *JAMA Network Open.*

Analysis	Outcome	Treatment	Factor	Subgroup	N_Placebo	N_Trt	N_events_Placebo	N_events_Trt	HR (95% CI)
PP	All cause	HCQ	Diabetes	N	163	159	7	6	0.86 [0.29; 2.57]
PP	All cause	HCQ	Diabetes	Y	45	38	1	4	4.90 [0.55; 43.88]
PP	All cause	HCQ	Cardiac Disease	N	106	102	2	4	2.09 [0.38; 11.41]
PP	All cause	HCQ	Cardiac Disease	Y	102	96	6	6	1.05 [0.34; 3.24]
PP	All cause	HCQ	Lung Disease	N	189	176	7	10	1.53 [0.58; 4.02]
PP	All cause	HCQ	Lung Disease	Y	19	22	1	0	0.00 [0.00; Inf]
PP	All cause	HCQ	Symptom Onset	< 120 hours	36	34	0	2	640189945.41 [0.00; Inf]
PP	All cause	HCQ	Symptom Onset	>= 120 hours	172	164	8	8	1.03 [0.39; 2.75]
PP	All cause	LPV/r	Age	<50	66	75	2	3	1.31 [0.22; 7.85]
PP	All cause	LPV/r	Age	>=50	137	119	6	13	2.56 [0.97; 6.72]
PP	All cause	LPV/r	Sex	F	110	110	4	4	1.00 [0.25; 3.98]
PP	All cause	LPV/r	Sex	M	98	90	4	12	3.39 [1.09; 10.51]
PP	All cause	LPV/r	Diabetes	N	163	161	7	11	1.60 [0.62; 4.12]
PP	All cause	LPV/r	Diabetes	Y	45	38	1	5	6.19 [0.72; 52.96]
PP	All cause	LPV/r	Cardiac Disease	N	106	91	2	6	3.56 [0.72; 17.65]
PP	All cause	LPV/r	Cardiac Disease	Y	102	109	6	10	1.57 [0.57; 4.31]
PP	All cause	LPV/r	Lung Disease	N	189	187	7	14	2.04 [0.82; 5.05]
PP	All cause	LPV/r	Lung Disease	Y	19	13	1	2	3.03 [0.27; 33.41]
PP	All cause	LPV/r	Symptom Onset	< 120 hours	36	30	0	2	738063520.52 [0.00; Inf]
PP	All cause	LPV/r	Symptom Onset	>= 120 hours	172	170	8	14	1.78 [0.75; 4.25]

© 2021 Reis G et al. *JAMA Network Open.*

Viral suppression analyses

eTable 3: Summary of Mixed-Effect Logistic Regression Model for Viral Suppression

Subset	Variable	Estimate	Std. Error	z value	Pr(>|z|)
Intention to treat analysis					
HCQ	(Intercept)	-3.033	0.591	-5.131	0.000
HCQ	Time	0.363	0.056	6.532	0.000
HCQ	Time:TreatmentHCQ	-0.094	0.059	-1.608	0.108
LPV/r	(Intercept)	-2.147	0.494	-4.348	0.000
LPV/r	Time	0.323	0.049	6.598	0.000
LPV/r	Time:TreatmentLPV/r	0.042	0.054	0.786	0.432
Per protocol analysis					
HCQ	(Intercept)	-3.234	0.590	-5.481	0.000
HCQ	Time	0.349	0.054	6.500	0.000
HCQ	Time:TreatmentHCQ	-0.024	0.057	-0.418	0.676
LPV/r	(Intercept)	-2.105	0.537	-3.923	0.000
LPV/r	Time	0.315	0.050	6.254	0.000
LPV/r	Time:TreatmentLPV/r	0.013	0.057	0.232	0.816

© 2021 Reis G et al. *JAMA Network Open.*

eTable 4: Summary of Sub-Group Analyses on Viral Suppression, Intention to Treat Analysis

Treatment	Factor	Subgroup	N_Placebo	N_Trt	OR (95% CI)
HCQ	Age	<50	72	77	0.98 [0.81; 1.18]
HCQ	Age	>=50	150	133	0.88 [0.76; 1.02]
HCQ	Sex	F	121	122	0.90 [0.77; 1.05]
HCQ	Sex	M	106	92	0.92 [0.78; 1.09]
HCQ	Diabetes	N	180	173	1.00 [0.91; 1.10]
HCQ	Diabetes	Y	47	40	0.24 [0.14; 0.43]
HCQ	Cardiac Disease	N	118	111	1.01 [1.01; 1.01]
HCQ	Cardiac Disease	Y	109	103	0.94 [0.75; 1.18]
HCQ	Lung Disease	N	207	190	0.86 [0.76; 0.98]
HCQ	Lung Disease	Y	20	24	4.92 [2.33; 10.40]
HCQ	Symptom Onset	< 120 hours	40	37	0.90 [0.69; 1.18]
HCQ	Symptom Onset	>= 120 hours	187	177	0.91 [0.80; 1.04]
LPV/r	Age	<50	72	89	1.10 [0.90; 1.33]
LPV/r	Age	>=50	150	149	1.02 [0.89; 1.16]
LPV/r	Sex	F	121	134	1.05 [0.90; 1.21]
LPV/r	Sex	M	106	110	1.03 [0.88; 1.21]
LPV/r	Diabetes	N	180	200	1.06 [0.94; 1.19]
LPV/r	Diabetes	Y	47	43	1.00 [0.78; 1.27]
LPV/r	Cardiac Disease	N	118	111	0.98 [0.84; 1.15]
LPV/r	Cardiac Disease	Y	109	133	1.08 [0.93; 1.26]
LPV/r	Lung Disease	N	207	229	1.03 [0.93; 1.15]
LPV/r	Lung Disease	Y	20	15	4.75 [2.29; 9.84]
LPV/r	Symptom Onset	< 120 hours	40	34	0.88 [0.25; 3.09]
LPV/r	Symptom Onset	>= 120 hours	187	210	1.06 [0.95; 1.19]
HCQ	Age	<50	66	72	0.83 [0.64; 1.07]
HCQ	Age	>=50	137	122	1.02 [0.92; 1.14]
HCQ	Sex	F	110	110	0.97 [0.83; 1.13]
HCQ	Sex	M	98	88	0.98 [0.83; 1.15]
HCQ	Diabetes	N	163	159	0.95 [0.83; 1.07]
HCQ	Diabetes	Y	45	38	1.10 [0.84; 1.44]
HCQ	Cardiac Disease	N	106	102	0.94 [0.79; 1.11]
HCQ	Cardiac Disease	Y	102	96	1.00 [0.86; 1.17]
HCQ	Lung Disease	N	189	176	0.96 [0.86; 1.08]
HCQ	Lung Disease	Y	19	22	1.15 [0.69; 1.93]
HCQ	Symptom Onset	< 120 hours	36	34	0.22 [0.14; 0.36]
HCQ	Symptom Onset	>= 120 hours	172	164	1.01 [0.92; 1.11]
LPV/r	Age	<50	66	75	1.02 [0.85; 1.23]
LPV/r	Age	>=50	137	119	1.01 [0.87; 1.17]
LPV/r	Sex	F	110	110	1.05 [0.90; 1.22]
LPV/r	Sex	M	98	90	0.98 [0.86; 1.11]
LPV/r	Diabetes	N	163	161	0.95 [0.84; 1.08]
LPV/r	Diabetes	Y	45	38	1.35 [0.96; 1.90]
LPV/r	Cardiac Disease	N	106	91	1.09 [0.92; 1.29]
LPV/r	Cardiac Disease	Y	102	109	0.95 [0.81; 1.11]
LPV/r	Lung Disease	N	189	187	1.03 [0.91; 1.15]
LPV/r	Lung Disease	Y	19	13	0.96 [0.52; 1.77]
LPV/r	Symptom Onset	< 120 hours	36	30	1.07 [0.80; 1.44]
LPV/r	Symptom Onset	>= 120 hours	172	170	1.01 [0.89; 1.14]

© 2021 Reis G et al. *JAMA Network Open*.

Symptoms analyses

eTable 5: Summary of Cox Proportional Hazard Regression Model for Time to COVID-19 Related Symptoms Resolution

Analysis	Treatment	HR (95% CI)	exp(coef)	se(coef)
ITT	HCQ	0.96 [0.76; 1.22]	0.960	0.120
ITT	LPV/r	0.88 [0.70; 1.12]	0.883	0.121
PP	HCQ	0.94 [0.74; 1.19]	0.936	0.123
PP	LPV/r	0.87 [0.68; 1.12]	0.873	0.127

© 2021 Reis G et al. *JAMA Network Open.*
