# Supplementary material for: Effect of Early Treatment With Hydroxychloroquine or Lopinavir and Ritonavir on Risk of Hospitalization Among Patients With COVID-19: The TOGETHER Randomized Clinical Trial
Source: JAMA Netw Open. 2021 Apr 22;4(4):e216468. doi: 10.1001/jamanetworkopen.2021.6468 (PMC8063069; doi:10.1001/jamanetworkopen.2021.6468)
Supplement: Supplement 3. — Data Sharing Statement [file jamanetwopen-e216468-s003.pdf]

Data Sharing Statement

Reis G, Moreira Silva EAS, Medeiros Silva DC, et al; the TOGETHER Investigators. Effect of early treatment with hydroxychloroquine or lopinavir and ritonavir on risk of hospitalization among patients with COVID-19: the TOGETHER randomized clinical trial. *JAMA Netw Open*. 2021;4(4):e216468. doi:10.1001/jamanetworkopen.2021.6468

Data
Data available: Yes
Data types: Deidentified participant data, Data dictionary
How to access data: By email request to the Principal Investigators: Dr. Edward Mills or Dr. Gilmar Reis
When available: With publication

Supporting Documents
Document types: Statistical/analytic code, Informed consent form
How to access documents: By email request to the Principal Investigators: Dr. Edward Mills or Dr. Gilmar Reis
When available: With publication

Additional Information
Who can access the data: Dr Edward Mills and Gilmar Reis
Types of analyses: Secondary analyses with analysis plans made available to and approved by the Principal Investigators
Mechanisms of data availability: With Investigator support and a signed data access agreement.
